# Influence of environmental, geographic, socio-demographic, and epidemiological factors on presence of malaria at the community level in two continents

**DOI:** 10.1038/s41598-024-67452-5

**Published:** 2024-07-20

**Authors:** Oswaldo C. Villena, Ali Arab, Catherine A. Lippi, Sadie J. Ryan, Leah R. Johnson

**Affiliations:** 1 The Earth Commons Institute, Georgetown University, Washington, DC 20057 USA; 2 Department of Mathematics and Statistics, Georgetown University, Washington, DC 20057 USA; 3 Department of Geography, University of Florida, Gainesville, FL 32611 USA; 4 Emerging Pathogens Institute, University of Florida, Gainesville, FL USA; 5 School of Life Sciences, University of KwaZulu-Natal, Durban, South Africa; 6 Department of Statistics, Virginia Tech, Blacksburg, VA 24061 USA; 7 Computational Modeling and Data Analytics, Virginia Tech, Blacksburg, VA 24061 USA; 8 Department of Biology, Virginia Tech, Blacksburg, VA 24061 USA

**Keywords:** Malaria, Vector-borne diseases, Malaria survey data, Temperature, Bioclimatic variables, *Plasmodium falciparum*, *Plasmodium vivax*, Africa, Asia, Computational biology and bioinformatics, Ecology, Climate sciences, Ecology, Environmental sciences, Diseases

## Abstract

The interactions of environmental, geographic, socio-demographic, and epidemiological factors in shaping mosquito-borne disease transmission dynamics are complex and changeable, influencing the abundance and distribution of vectors and the pathogens they transmit. In this study, 27 years of cross-sectional malaria survey data (1990–2017) were used to examine the effects of these factors on *Plasmodium falciparum* and *Plasmodium vivax* malaria presence at the community level in Africa and Asia. Monthly long-term, open-source data for each factor were compiled and analyzed using generalized linear models and classification and regression trees. Both temperature and precipitation exhibited unimodal relationships with malaria, with a positive effect up to a point after which a negative effect was observed as temperature and precipitation increased. Overall decline in malaria from 2000 to 2012 was well captured by the models, as was the resurgence after that. The models also indicated higher malaria in regions with lower economic and development indicators. Malaria is driven by a combination of environmental, geographic, socioeconomic, and epidemiological factors, and in this study, we demonstrated two approaches to capturing this complexity of drivers within models. Identifying these key drivers, and describing their associations with malaria, provides key information to inform planning and prevention strategies and interventions to reduce malaria burden.

## Introduction

Malaria is the deadliest vector-borne disease worldwide, causing 249 million infections and taking the lives of 608,000 people in 2022 alone^1^. The overwhelming majority (94%) of malaria cases occur in Africa, where *Plasmodium falciparum* is the most prevalent malaria parasite, accounting for 99.5% of cases versus the 0.5% caused by *P. vivax*^1^. Asia also has considerable malaria burden, where 50.9% of cases are caused by *P. vivax* and 49.1% are caused by *P. falciparum*^2^. Despite a 14-year decline in global malaria cases and deaths following continued intervention efforts, a major resurgence of malaria has occurred worldwide since 2014^1,3,4^. One of the biggest challenges to reducing malaria burden is understanding the complex interaction of factors that drive and shape malaria transmission dynamics.

Transmission dynamics of mosquito-borne diseases (MBDs), like malaria, can be difficult to describe and predict due to multiple levels of complexity arising from interactions between vectors, pathogens, hosts, and the environment^5,6^. Transmission of MBDs is directly impacted by climate, particularly temperature and precipitation, and to a lesser degree humidity and wind patterns^7,8^. Yet, there are many other factors that can mediate the spatial and temporal distribution, intensity, and duration of MBDs^5,6,9^. There is evidence to support that malaria occurrence, transmission, and seasonality are influenced by environmental (e.g., isothermality), socio-economic (e.g., human population density), and health factors (e.g., access to health services)^10^. Yet, the influence of these interacting elements on malaria transmission dynamics is often not well understood, or are highly variable across regions.

The effects of temperature and rainfall on malaria dynamics are probably the most well studied because these two factors are believed to have the greatest direct impact on mosquito-borne diseases^9^. Mosquitoes are ectotherms, and temperature affects their physiology, behavior, and development^11^. Furthermore, temperature also affects the development of *Plasmodium* parasites inside the mosquito (e.g., the extrinsic incubation period,^12^). Therefore, malaria transmission dynamics are highly constrained by temperature^11,13–16^. Precipitation and related factors (e.g., evaporation rates, presence of breeding habitat, irrigation, etc.) also play vital roles in MBD transmission dynamics by creating habitats necessary for the aquatic stages of mosquito development^9^, thereby impacting vector abundance and distribution^17–19^.

Although temperature and precipitation are key drivers of MBDs, additional factors affect malaria transmission dynamics, but are less well-studied. These include heterogeneous human population density linked to urbanization, economic development (e.g., measured as gross domestic product per capita, GDPPC, or the human development index, HDI or habitat which can be measured as variables such as normalized difference vegetation index (NDVI) or elevation). For example, prior studies found human population density to be a reliable metric to define patterns of malaria risk, with moderately populated areas typically having high malaria prevalence^20,21^. In Africa, malaria was not historically considered a public health problem in urban centers, compared to rural areas, because urbanization is associated with the reduction of suitable breeding habitats and vegetation cover for the primary malaria vectors, *Anopheles gambiae*, *An. arabiensis*, An. coluzzii, and *An. Funestus*^22,23,24]﻿^. In contrast, *An. stephensi*, *An. minimus*, and *An. dirus* are the main malaria vectors in southern and western Asia, where urban and coastal malaria outbreaks are more common^24^. However, the recent expansion of *An. stephensi* from Asia into Africa^25^ poses a major potential health risk for densely populated urban areas in Africa, as this malaria vector is well adapted to reproduce in built environments^26^. Changes in temperature and precipitation patterns due to climate change has significant implications for malaria vectors and the transmission dynamics of malaria such as vector survival and development, parasite development, range expansion, length of transmission season, availability of breeding sites, and insecticide resistance, etc.^27–29^.

Poverty is historically associated with malaria since families in areas with lower GDPPC usually have less access to quality housing, health services, and municipal water and sanitation services^30,31^. In the past, environmental and socio-economic changes contributed to malaria eradication in the USA and Europe, and improved control in most of Central and South America^32,33^. However, the relationship between GDPPC and malaria may be bi-directional: GDPPC could affect malaria prevalence and/or malaria prevalence could affect countries’ GDPPC. Similarly, lower values of HDI are associated with high malaria^34^. Measures of environmental conditions can also serve as predictors of malaria activity. While there is consensus that high malaria prevalence occurs in areas of moderate elevation, the utility of other indicators, like NDVI, can vary across studies^21^. Some studies have found that NDVI is positively associated with mosquito abundance, mosquito community assembly, and malaria cases^35,36^. However, other studies found that NDVI is negatively associated with malaria cases^37^. These discrepancies could be due to the differences in the underlying habitat preferences of the mosquitoes, perhaps making NDVI useful in more nuanced modeling applications^37^.

In this study, an integrated modeling framework for evaluating impacts of environmental, geographic, socio-demographic, and epidemiological factors on malaria in Sub-Saharan Africa and Southeast Asia is presented and implemented. Additionally, the association of *P. falciparum* and *P. vivax* malaria survey data with the basic reproductive number (*R*_0_), defined as the number of secondary cases that on average an infected individual will cause in a susceptible population, was assessed. To our knowledge, this is the first study to incorporate *R*_0_ as a factor to assess for its association with malaria survey data.

## Methods

### Malaria survey data

Data on *P. falciparum* and *P. vivax* malaria at the community level in 46 countries in Africa and 21 countries in Asia from 1990 to 2017 (Figure S1; supplemental material) were obtained from the Malaria Atlas Project (MAP), https://malariaatlas.org/^38,39^. The survey-data collected by MAP followed the General Data Protection Regulation (GDPR) and associated data protection legislation, https://malariaatlas.org/privacy-policy/. The survey data is publicly available and consist of the number of individuals at each sampled location (i.e., longitude, latitude) observed to have *P. falciparum* and *P. vivax* parasites in their blood, together with the total number of individuals sampled, respectively. Data was aggregated from multiple malaria survey studies which number varies by community, country, and continent. The malaria survey data were converted to presence/absence of malaria at the community level (i.e., 1 was assigned if malaria was present and 0 is malaria was absent). *Plasmodium vivax* malaria in Africa was not assessed due to insufficient data (718 records of which only 155 showed malaria presence; Table 1).

**Table 1:** Absence and presence of malaria by parasite and by continent at the community level aggregated from the Malaria Atlas Project.

Parasite	Continent	Number of communities	Absence (0)	Presence (1)
*P. falciparum*	Africa	5376	1970	5810
*P. vivax*	Africa	574	563	155
*P. falciparum*	Asia	2276	1314	1453
*P. vivax*	Asia	2438	1604	1281

Note that these data are aggregated from multiple survey studies, many of which were designed specifically to capture malaria incidence. They are not distributed equally in space or time, and often exclude sampling from areas with a priori low expected malaria.

### Environmental and geographic variables

#### Temperature and precipitation

Monthly temperature and precipitation data from 1990 to 2017 were obtained from the WorldClim Global Climate Data Project using the *raster* package in R^40^, at a 5-min spatial resolution (17.3 km^2^). Aggregated mean temperature was calculated for the two quarters (three calendar months) prior to the start month of each survey study (Table 2). Isothermality (bio3), a bioclimatic variable from the WorldClim project which quantifies how much day-to-night temperatures oscillate relative to the summer-to-winter annual oscillations, was also considered^41^. Average monthly precipitation (mm) was aggregated to mean precipitation for two quarters prior to the start month of each survey study. Two additional bioclimatic variables were also considered: precipitation of the wettest quarter (bio16), which is the total precipitation for all three months with the highest cumulative precipitation; and the precipitation of the driest quarter (bio17)^40^.

**Table 2:** List of the factors used to assess the effect of environmental, geographic, socio-demographic, and epidemiological factors on malaria.

Variable	Unit	Temporal resolution	Source
*Plasmodium falciparum* survey data	Individuals	2 Months frame	Malaria Atlas Project (MAP)
*Plasmodium vivax* survey data	Individuals	2 Months frame	Malaria Atlas Project (MAP)
Average temperature of previous quarter	_°C_	Quarter—yearly	WorldClim project
Average temperature before previous quarter	_°C_	Quarter—yearly	WorldClim project
Average precipitation of previous quarter	mm	Quarter—yearly	WorldClim project
Average precipitation before previous quarter	mm	Quarter—yearly	WorldClim project
Isothermality (bio3)	%	Yearly	WorldClim project
Precipitation of wettest quarter (bio16)	mm	Quarter-yearly	WorldClim project
Precipitation of driest quarter (bio17)	mm	Quarter-yearly	WorldClim project
Year of the survey study	Year	Yearly	Malaria Atlas Project (MAP)
Gross domestic product per capita (GDPPC)	Dollars	Yearly	(Kummu et al.^47^)
Human development index (HDI)	0 to 1	Yearly	(Kummu et al.^47^)
Population density	People/Km^2^	Yearly	Global Rural–Urban Mapping Project (GRUMP)
Elevation	Meters	30arc-seconds	United States Geological Survey—USGS
Normalized Difference Vegetation Index (NDVI)	0 to 1	Monthly	EARTHDATA project, NASA and USGS
Basic reproductive number of previous quarter (*R*_0_q1)	0 to 1	Two months frame	(Villena et al.^15^)
Basic reproductive number before previous quarter (*R*_0_q2)	0 to 1	Two months frame	(Villena et al.^15^)

Two dependent variables: (1) *Plasmodium falciparum* survey data and (2) *Plasmodium vivax* survey data and 15 predictor variables. The spatial resolution for all these variables is at the community level.

#### Elevation and normalized difference vegetation index

For topographical data, the Global Multi-resolution Terrain Elevation Data 2010 (GMTED2010) from the U.S. Geological Survey (USGS), with a horizontal grid spacing of 30 arc-seconds (approximately 1 km) was used. Normalized Difference Vegetation Index (NDVI) data, a greenness measure estimating chlorophyll density in vegetation cover, were obtained from the Famine Early Warning Systems Network (FEWS-NET). FEWS-NET NDVI data were generated from the collection of Moderate Resolution Imaging Spectroradiometer (MODIS) instruments flown aboard the Aqua satellite from July 2002 to current times^42^. Real-time and historical NDVI data are composited in 10-day (dekadal) intervals at 250 m spatial resolution^43^. For the studies conducted from 2000 to July 2002, we used MODIS vegetation indices (MOD13A3) collection 6 at 1 km (km) spatial resolution from the EARTHDATA project from USGS and NASA, available from 2000 onward. For malaria survey data prior to 2000, a monthly average NDVI derived from EARTHDATA from 2000 to 2004 was calculated using the “Cell Statistics” function in ArcGIS 10.8.1^44^.

#### Socio-demographic variables

The socio-demographic variables used were population density, gross domestic product per capita (GDPPC), and the human development index (HDI) at the community level (e.g., where the individual survey studies took place).

Population density was estimated at the site level where malaria surveys occurred. Population density data were obtained from the Gridded Population of the World (GPW) collection from the Global Rural–Urban Mapping Project—(GRUMP) project^45^. We used GRUMP global population density at five-year intervals from 1990 to 2015 (i.e., 1990, 1995, 2000) with a resolution of 30 arc-seconds (1 km). We extracted population density data in intervals of 5 years (e.g., from the 1990 raster data set we extracted approximate population density from 1990 to 1994). Due to high variability in population density across survey study locations, in our models, we scaled population density using the “scale” function in R^46^ where the vector mean is subtracted from each *x*_*i*_ value and divided by the standard deviation of the vector.

Gross Domestic Product per capita (GDPPC) and HDI data from 1990 to 2015 were obtained from the Gridded Global Datasets^47^. The GDPPC data indicate the purchasing power parity in constant 2011 international US dollars. The HDI data represent key aspects of development, namely life expectancy, education expressed as years of schooling, and per capita income indicators. The GDPPC and the HDI data are available at the sub-national level for the whole world at 5 arc-min resolution and WGS84 projection^47^. GDPPC and HDI data were in the form of network common data form (NetCDF), which were converted to raster using the “Make NetCDF Raster Layer” tool from ArcGIS 10.8.1.

The raster data sets (i.e., temperature, precipitation, elevation, NDVI, population density, GDPPC, HDI) which spans different spatial and temporal resolutions were imported into ArcGIS version 10.8.1 and resampled if needed and synchronized to the timing of the response variable. We resampled the raster datasets using either the *nearest* method for variables like NDVI or the *bilinear* method for layers with continuous data^48,49^. Next, data for each survey study site was extracted using geographic coordinates (latitude and longitude) as a merging points employing the “extract values to points” tool from the spatial analysis toolset in ArcMap^50^. For NDVI, we used bilinear interpolation which means that the value of the cell was calculated from the adjacent cells^51^.

#### Epidemiological component

The basic reproductive number *R*_0_ of each malaria species (i.e., *P. falciparum*) and region (i.e., Africa) was also used as a predictor variable. *R*_0_, the average number of secondary cases that one infected individual generates during an infectious period in a susceptible population, was estimated for each *Anopheles*-pathogen pairing in a previous study by Villena et al.^15^. They used the most common parameterization of *R*_0_ for vector-borne infections which is based on the Ross-MacDonald model of malaria transmission^52^. More specifically, they incorporated multiple temperature dependent mosquito and parasite traits^11,13,14,53^ into the following equation:

1 $${S\left(T\right)= \left(\frac{{a\left(T\right)}^{2}bc\left(T\right){e}^{\frac{-\mu \left(T\right)}{PDR(T)}}EFD\left(T\right){P}_{EA}\left(T\right)MDR(T)}{{\mu (T)}^{3}}\right)}^\frac{1}{2}$$

where *a* is the mosquito biting rate; *bc* is vector competence, which is a combination of *b*, the probability of a person becoming infected by a bite of an infected mosquito, and *c*, the probability of a vector becoming infected by feeding on an infectious person; µ is the mosquito mortality rate; *PDR* is the parasite development rate; *EFD* is the mosquito fecundity expressed as the number of eggs per female per day; *PEA* is the proportion of eggs surviving to adulthood; *MDR* is the mosquito development rate^15^. All mosquito and malaria parasite traits were obtained under laboratory conditions and at constant temperatures^14,15^. *R*_0_ data were matched with aggregated mean temperatures for each of the two quarters prior to the start month of each malaria prevalence study, using temperature as a merging variable. Thus, *R*_0_, unlike many of the other predictors, corresponds to a study/location in both space and time.

#### Data analysis with CART and GLMs

Malaria presence/absence data and its relationship to multiple predictors was analyzed (Table 2) separately for *P. falciparum* and *P. vivax* for Africa and Asia (four subsystems). To do this, two different approaches were used: a classification and regression tree (CART) and a generalized linear model (GLM). The CART approach is a non-parametric statistical model applicable to both numerical and categorical data^54^. A GLM is a flexible generalization of ordinary linear regression that can be used to model data that are not normally distributed^55,56^. These two methods are widely used to assess the relationship between a continuous or categorical response variable and predictor variables^57^. Each method has its own advantages and disadvantages. For example, GLMs allow the use of the Bayes Information Criterion (BIC) and stepwise regression to automatically find the “best” model, can have non-linear model specifications, and can handle different response distributions. Some disadvantages of GLMs are the assumptions around the chosen statistical distribution of the response data (which can be restrictive), difficulty in finding a global best model for large predictor dimension, and sensitivity to outliers. Conversely, CART models make few assumptions about the nature of relationships, have no parametric assumptions, and allow for the analysis of many data types (e.g., continuous, binary, ordinal, nominal). Disadvantages of CART models include the lack of variables combinations in each split, potentially unstable tree structures (e.g., change in the sample may give different trees), and focus of optimization at each split (e.g., solutions may not be globally optimal)^57,58^.

For the CART approach the *rpart* function from the *rpart* (Recursive partioning and regression trees) package was used^59^ in R^46^. The *rpart* function constructs a CART by splitting the dataset and fitting a constant model (here, estimated proportion) within each subset (i.e., at each leaf). Splits are recursive, so that the subsets resulting from a split are further split until a predetermined termination criterion is reached. More specifically, at each step, the split is chosen to occur on the independent variable that results in the largest possible reduction of heterogeneity of the dependent variable, until an impurity state of zero (i.e., the class is homogeneous) or close to zero is reached^60^. The Gini index was used to quantify the level of impurity in our CART model fits, where the Gini index reaches maximum value when all classes in the table have equal probability^60^. The trees generated in this study were built using the following process: first a single variable is found which best splits the data in two groups (i.e., child nodes). The process is then applied separately to each subgroup, and so on recursively until the subgroups either reach a minimum size (100 for these data) or until no improvement can be made.

For the GLM approach, each observation at each location in our dataset was categorized with a binary response variable to indicate whether or not malaria had been observed in sampled individuals in our malaria survey dataset. That is, the response *y*_*i,j*_ was defined for the *j*^th^ individual in location *i* as

2 $${y}_{i,j}=\left\{\begin{array}{*{20}l} {1,}  & {if\:an\:observed\:community\:at\:location\:i\: presents\:malaria\:cases}\\ {0,} &  {otherwise}\end{array}\right.$$

The general logistic GLM is defined by the mean equation:

3 $$Pr\left( {y_{i,j} = \, 1} \right) \, = \theta = \, logit^{ - 1} \left( {\eta_{i,j} } \right)$$

4 $$\eta_{i,j} = \beta_{0} + \beta_{1} x_{1,i} + \beta_{2} x_{2,i} + ... + \beta_{n} x_{n,I}$$

where *η*_*i*_ is the linear predictor, with *β*_0_ as the intercept, *β*_1_,…., *β*_*n*_ are regression parameters, and *x*_1_*, . . . , x*_*n*_ are the location and time dependent explanatory variables. Observations at data point *i, j* are then Binomial random variables with “success” probability *θ*_*i*_ and sample size *N*_*i*_ at each location. We implemented the GLMs using the function *glm* in R^46^.

A null model, which estimated an intercept only (in effect estimating a single proportion for all sites) was fit, and a full model was also fit including the linear effects of: mean temperature for the two quarters prior to the start of the survey study; mean precipitation for the two quarters prior to the start of the survey study; isothermality (bio3); precipitation of the wettest (BIO 16) and driest (BIO 17) quarter; gross domestic product per capita (GDPPC); the human development index (HDI); population density; the temperature dependent basic reproductive number (*R*_0_) for the two previous quarters of the start of the survey study; year in which the survey study started (year); elevation (elev); and the normalized difference vegetation index (NDVI). A quadratic term for isothermality (BIO 3), temperature, and precipitation was also included in order to capture the non-linear response of these factors.

Next, forward stepwise variable selection was performed using the step function in R^46^ to choose a final best fitting model for comparison. Finally, to assess if model assumptions were adequately met, the randomized quantile residuals (RQRs) were computed and plotted, using the *statmod* package^61^ for each of the Null, full, and stepwise chosen models. RQRs are the residuals of choice for GLM models in large dispersion situations^62^.

#### Model assessment

CART and GLM performance were assessed by estimating the accuracy, precision, recall, and F1-score for each model^63–65^. Model accuracy is defined as the total correctly classified samples divided by the total number of classified samples. Model precision refers to the positive patterns that are correctly predicted from the total number of positive classified samples (true and false positives). Model recall is a measure of the fraction of positive patterns that are correctly classified. The F1-score is the weighted mean of the precision and the recall (See formulas in the section B.1.1; supplemental material)^64,66,67^. To estimate these performance metrics, the dataset was divided into a training and test dataset, using a stratified random sampling method which divides the dataset into smaller subgroups called strata. Strata are formed based on samples that share attributes or characteristics^68^. Our study dataset was grouped by malaria type (*P. falciparum* and *P. vivax*), continent, country, and the survey study year, then 70% of the data were randomly divided into the training set and remaining 30% to the testing set.

## Results

### *Plasmodium falciparum* malaria in Africa

**Figure 1:**
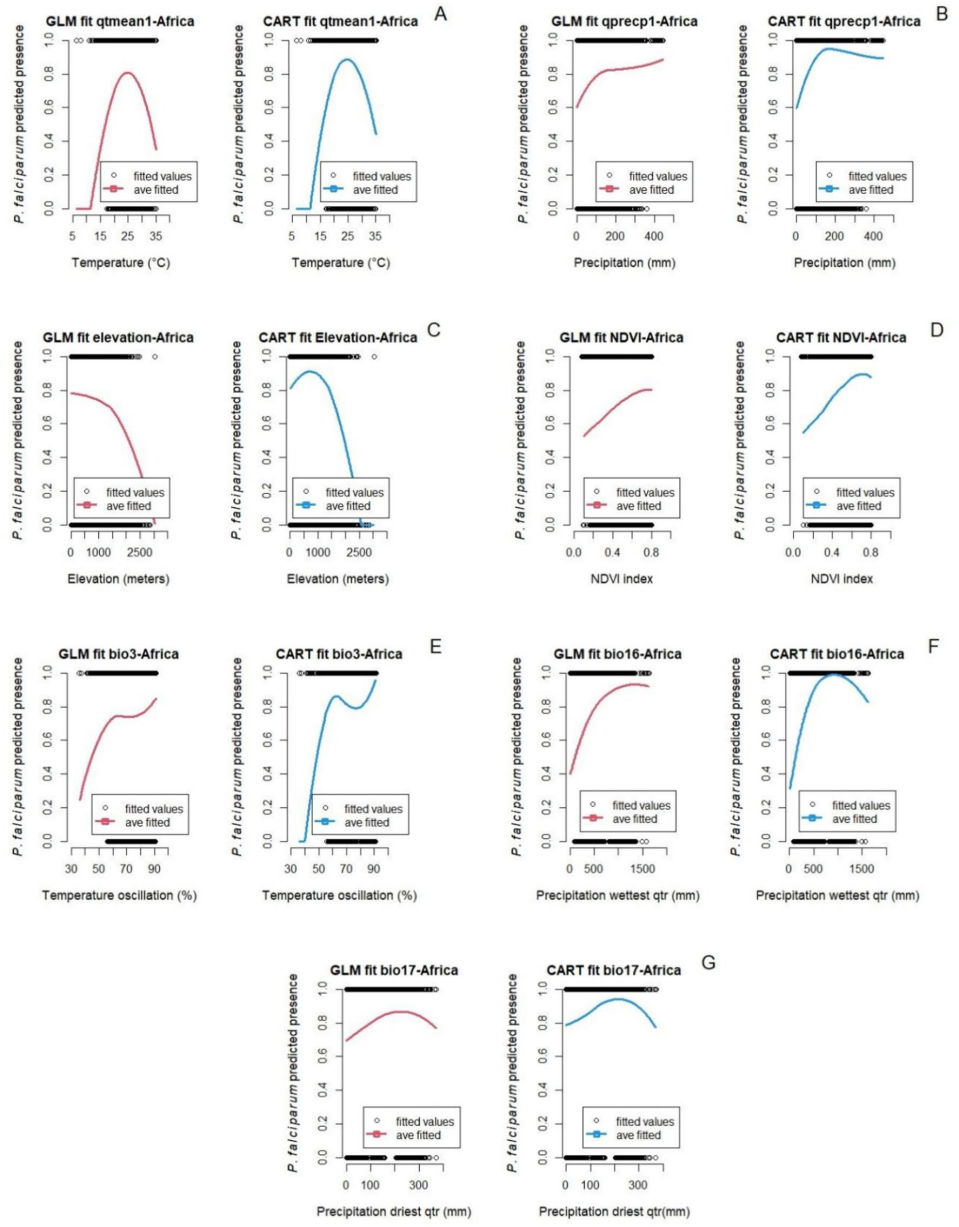
Marginal predictions based on particular environmental and bioclimatic predictors for *P. falciparum* in Africa. (**A**) Temperature 1st quarter prior to the start of the survey study, (**B**) Precipitation 1st quarter prior to the start of the survey study, (**C**) Elevation, (**D**) NDVI, (**E**) Isothermality, (**F**) Precipitation of the wettest quarter, and (**G**) Precipitation of the driest quarter.

**Figure 2:**
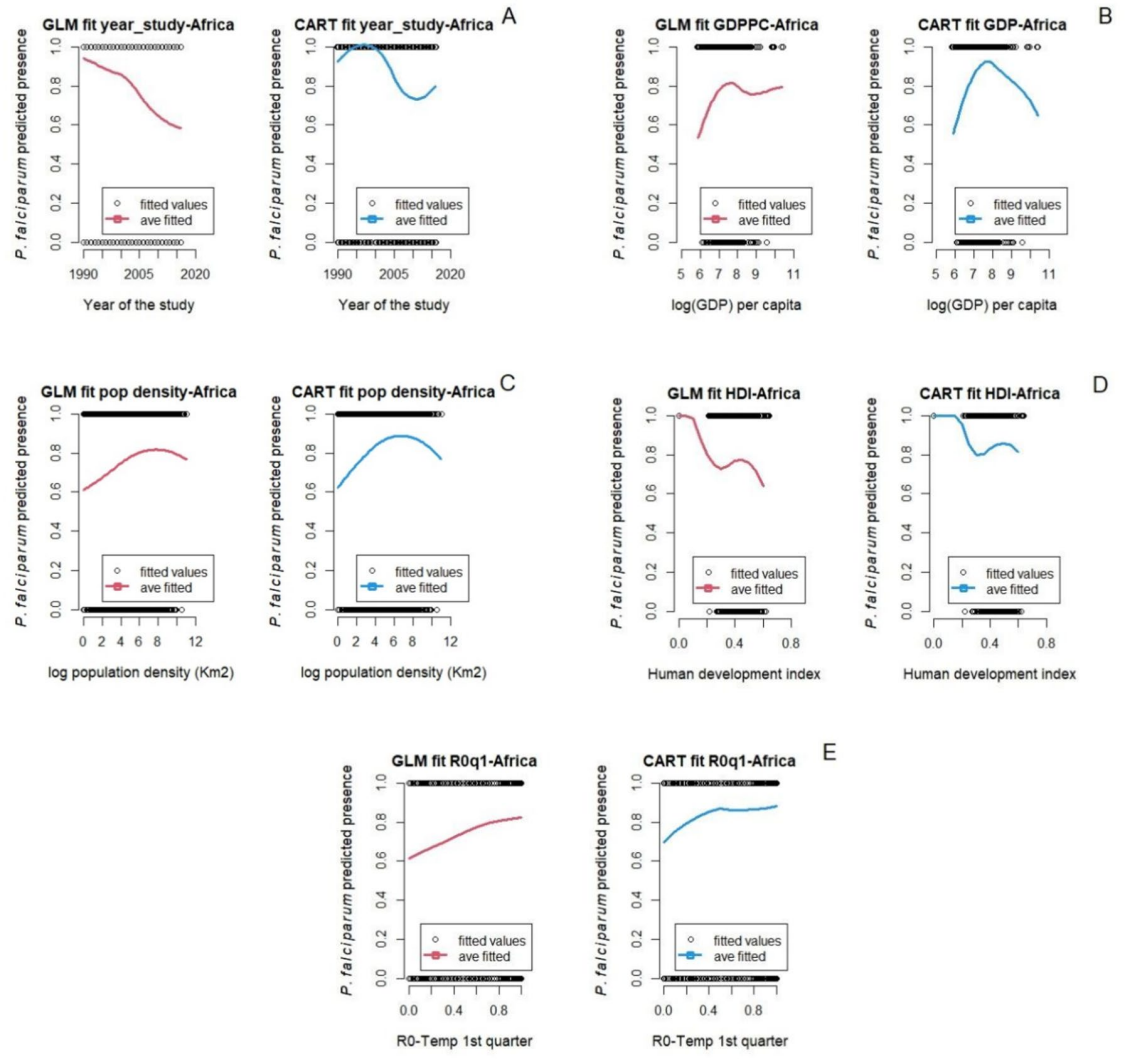
Marginal predictions based on socio-demographic and epidemiological predictors for *P. falciparum* in Africa. (**A**) Year at which the survey study started, (**B**) Gross domestic product per capita, (**C**) Population density, (**D**) Human development index, and (**E**) Basic reproductive number (*R*0) 1st quarter prior to the start of the survey study.

Figure 1 shows the fitted marginal relationships between *P. falciparum* malaria in Africa and environmental and bioclimatic factors in the quarter before each survey study period. Additional marginal predictions for earlier quarters are in supplementary material (Figure S2). Marginal predictions for *P. falciparum* malaria showed qualitatively similar responses for both GLM and CART models (Figs. 1 and 2), although the CART model had better accuracy, precision, and F1-score than the GLM (Table S7; supplemental material). For example, the temperature in the quarter prior to the *P. falciparum* malaria survey study at which presence is predicted to be maximized is 24.9 ^°^C (GLM model) and 24.8 ^°^C (CART model), decreasing on either side. The thermal range where predicted malaria presence is non-negligible, is between approximately 12 ^°^C and 36 ^°^C for both models (Fig. 1A). In contrast, marginal predictions for precipitation variables are not as consistent. Precipitation of the prior quarter and *P. falciparum* malaria showed a positive relationship up to approximately 150 mm of precipitation. After this point, the predicted relationship levels off in the GLM model, but decreases in the CART model (Fig. 1B). Isothermality (BIO 3) is unusual, showing a potentially higher order nonlinear relationship with *P. falciparum* malaria presence (e.g., possibly cubic, Fig. 1E).

The relationship between socio-demographic and epidemiological factors and *P. falciparum* malaria in Africa are shown in Fig. 2. While some predictions of the two models are again similar, they are less consistent than the patterns seen with the environmental predictors. For example, high *P. falciparum* malaria presence is seen from 1990 to 2000, and both the GLM and CART models capture the constant decline of *P. falciparum* from 2000 to 2012 well. However, after 2012, the GLM model continues to predict that *P. falciparum* malaria declines and whereas the CART model showed a resurgence of *P. falciparum* malaria (Fig. 2A). This is likely due to the additional flexibility available in the CART model (we only considered up to quadratic terms for the GLM). Predictions for malaria presence with per capita log GDP are similarly disparate between the two models. This may be because these predictors are correlated with each other, and the models separate out the effects of these correlated variables in different ways.

The pruned tree for *P. falciparum* malaria in Africa from the CART model is given in Supplemental Material (See section A.2), along with the coefficients, standard errors, and P-values from the GLM model (Table S1), a graph of the magnitude and uncertainty of the fitted parameter estimates of each variable of the GLM model (Figure S9), and the graphs for the randomized quantile residuals and the density plot from the GLM Model (Figures S12-A and S12-B).

### *Plasmodium falciparum* malaria in Asia

**Figure 3:**
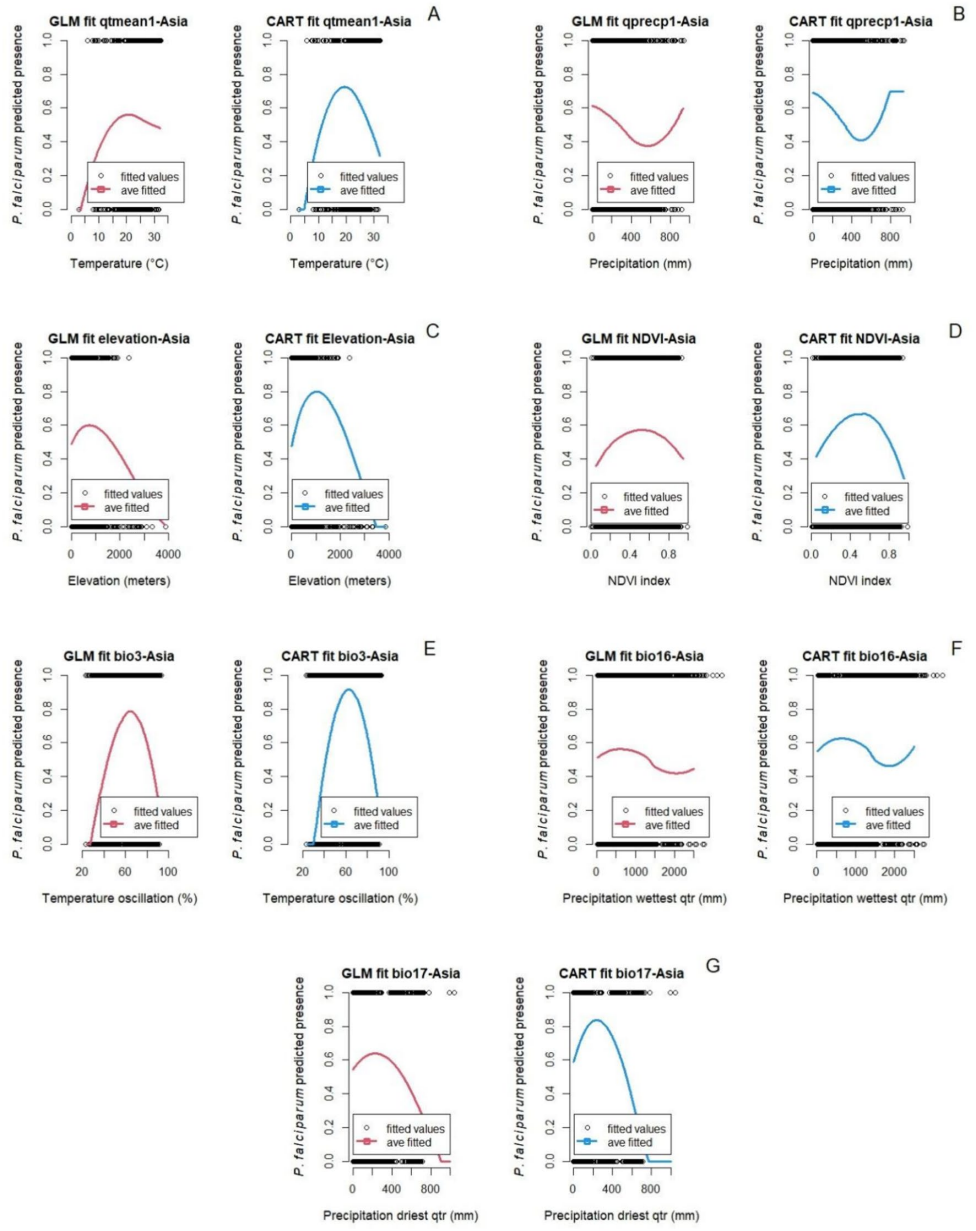
Marginal predictions based on particular environmental and bioclimatic predictors for *P. falciparum* in Asia. (**A**) Temperature 1st quarter prior to the start of the survey study, (**B**) Precipitation 1st quarter prior to the start of the survey study, (**C**) Elevation, (**D**) NDVI, (**E**) Isothermality, (**F**) Precipitation of the wettest quarter, and (**G**) Precipitation of the driest quarter.

**Figure 4:**
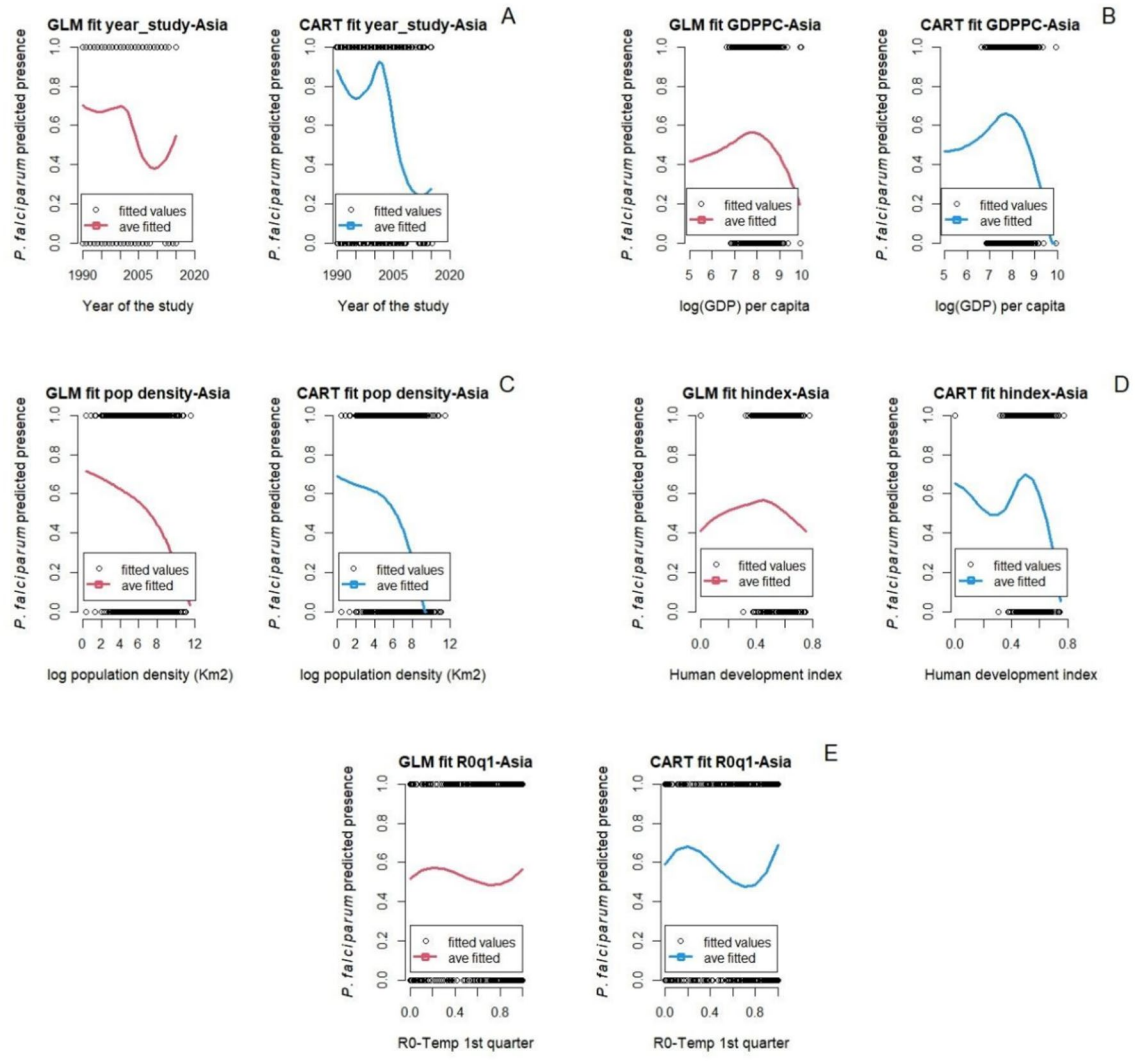
Marginal predictions based on socio-demographic and epidemiological predictors for *P. falciparum* in Asia. (**A**) Year at which the survey study started, (**B**) Gross domestic product per capita, (**C**) Population density, (**D**) Human development index, and (**E**) Basic reproductive number (*R*0) 1st quarter prior to the start of the survey study.

Figure 3 shows the fitted marginal relationships between *P. falciparum* malaria in Asia and environmental and bioclimatic factors in the quarter before each survey study period. Results from marginal predictions two quarters prior to the survey study are in the supplementary materials (Figure S3). Similar to *P. falciparum* malaria in Africa, marginal predictions for *P. falciparum* malaria in Asia showed similar responses for both GLM and CART (Figs. 3 and 4). For example, the normalized difference vegetation index (NDVI) showed a unimodal response: increasing *P. falciparum* malaria up to a NDVI *∼* 0.53 followed by a decrease (Fig. 3D). Similarly, Isothermality (BIO 3) is unimodal with a predicted peak in *P. falciparum* malaria when bio3 is around 62% (Fig. 3E). In contrast, marginal predictions related to temperature and elevation are not as consistent. The temperature in the quarter prior to the *P. falciparum* malaria survey study in Asia at which malaria presence is predicted to be maximal is 21 ^°^C (GLM) and 19.5 ^°^C (CART) (Fig. 3A), but the decline at higher temperatures is much less in the GLM than the CART fit. For elevation, the models predict maximum malaria presence when elevation is 752 m (GLM) versus 1,010 m (CART), with malaria decreasing at higher elevations, although again, at different rates (Fig. 3C).

In Fig. 4, the relationship between socio-demographic and epidemiological factors with *P. falciparum* malaria in Asia are shown. Again, predictions of the two models are similar, except for the predictions by year and the human development index. Similar to *P. falciparum* malaria predictions in Africa, high *P. falciparum* malaria from 1990 to 2001 is observed. After that, both the GLM and CART models capture a constant decline of *P. falciparum* malaria well until 2009 and 2011 for GLM and CART model respectively, then *P. falciparum* malaria shows a resurgence, although this effect is larger in the GLM (Fig. 4A). In the GLM model, the relationship between human development index (HDI) and *P. falciparum* malaria is hump-shaped, with a peak at *∼*0.44. However, the relationship in the CART model is instead higher order, showing an initial decline in malaria presence up to a HDI *∼* 0.265, then increasing until *∼*0.49, then decreasing again as the HDI increases further (Fig. 4D).

The pruned tree for *P. falciparum* malaria in Asia from the CART model is given in Supplemental Material (See section A.3), along with the coefficients, standard errors, and p-values from the GLM model (Table S2), a graph of the magnitude and uncertainty of the fitted parameter estimates of each variable of the GLM model (Figure S10), and the graphs for the randomized quantile residuals and the density plot from the GLM Model (Figures S12-C and S12-D).

### *Plasmodium vivax* malaria in Asia

**Figure 5:**
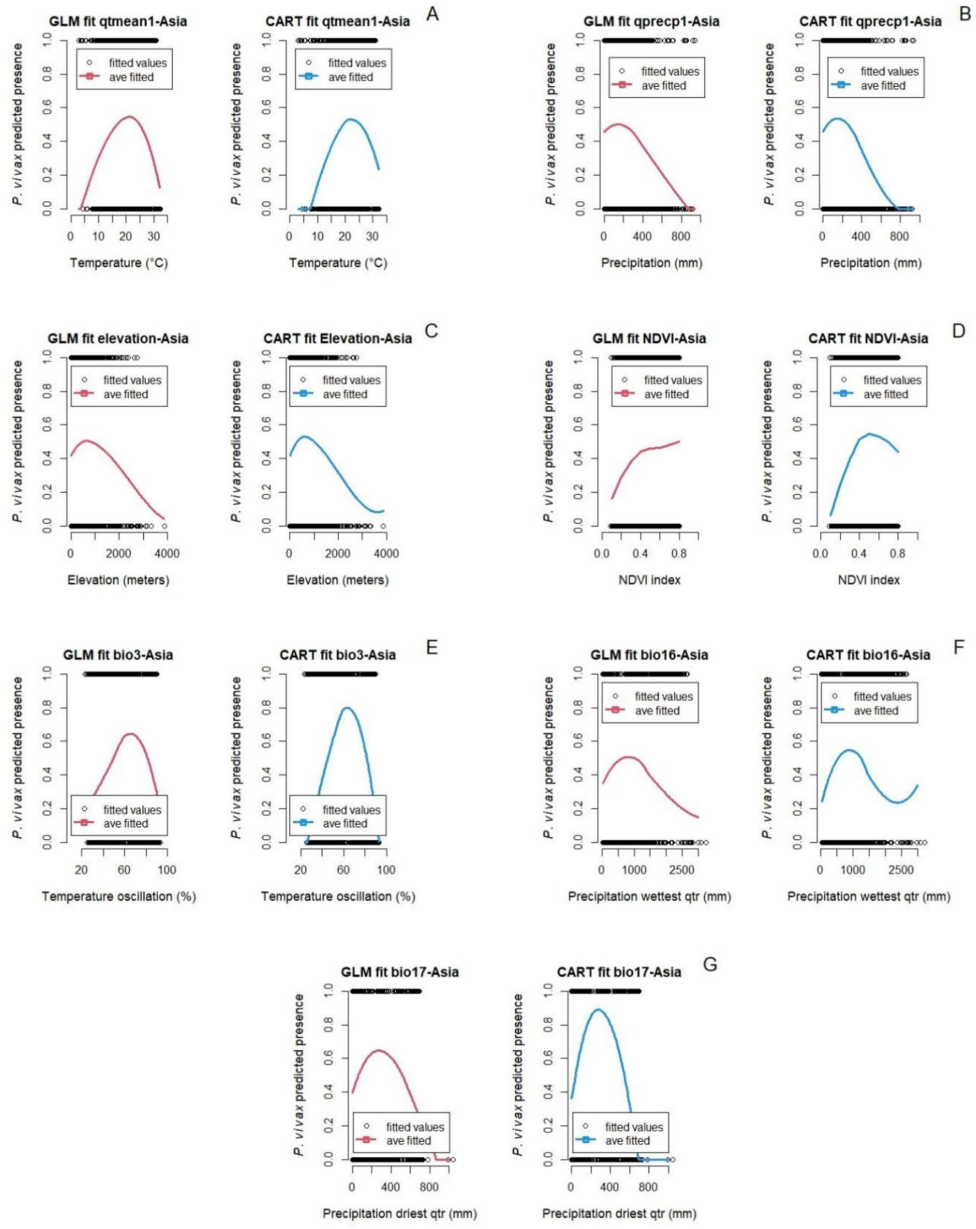
Marginal predictions based on particular environmental and bioclimatic predictors (**A**) Temperature 1st quarter prior to the start of the survey study, (**B**) Precipitation 1st quarter prior to the start of the survey study, (**C**) Elevation, (**D**) NDVI, (**E**) Isothermality, (**F**) Precipitation of the wettest quarter, and (**G**) Precipitation of the driest quarter for *P. vivax* in Asia.

**Figure 6:**
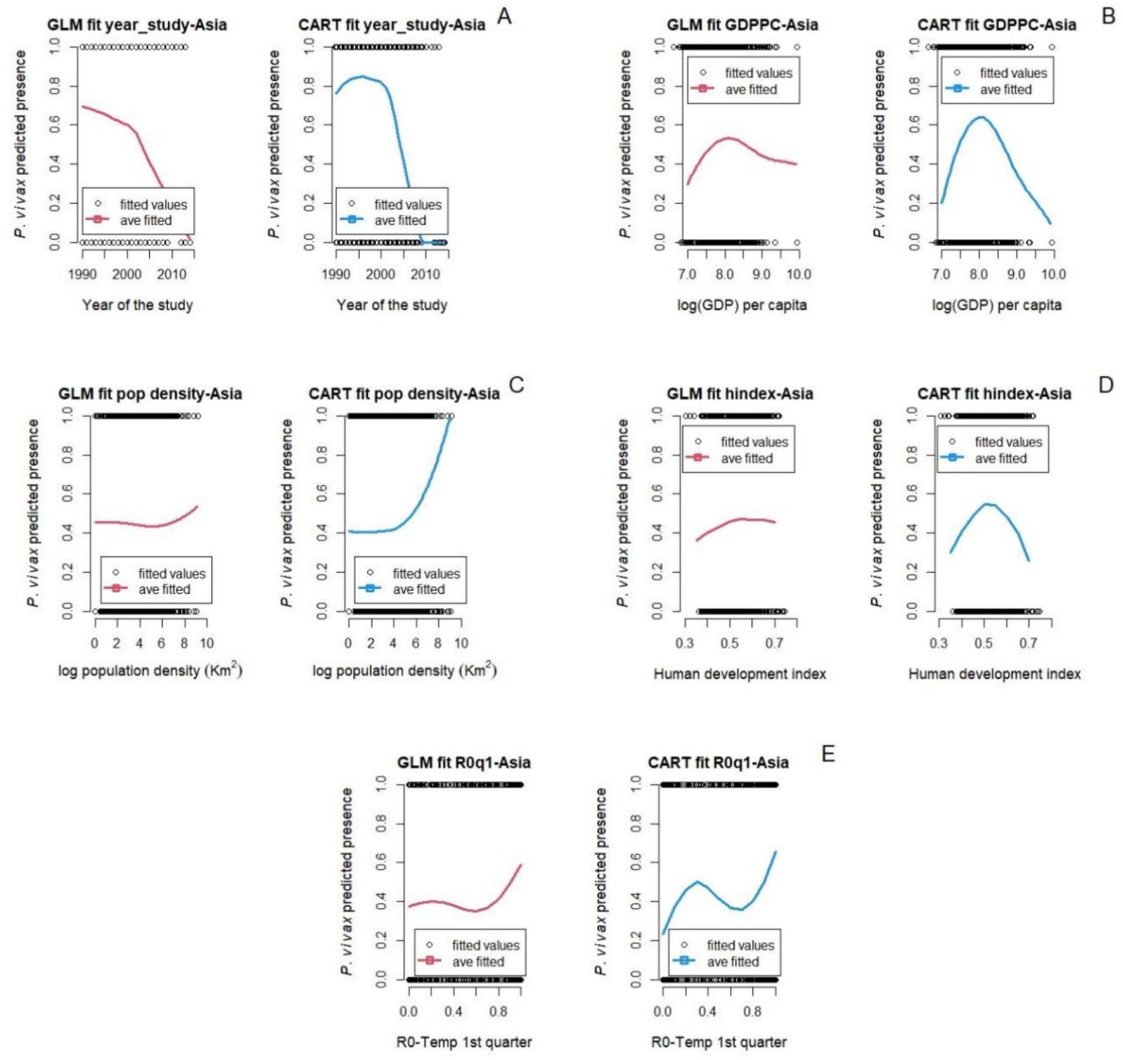
Marginal predictions based on socio-demographic and epidemiological predictors (**A**) Year at which the survey study started, (**B**) Gross domestic product per capita, (**C**) Population density, (**D**) Human development index, and (**E**) Basic reproductive number (*R*0) 1st quarter prior to the start of the survey study for *P. vivax* in Asia.

Figure 5 shows the fitted marginal relationships between *P. vivax* malaria in Asia and environmental and bioclimatic factors in the quarter before each survey study period. Additional results for marginal predictions in earlier quarters are in the supplementary materials (Figure S4). The CART model showed better accuracy, recall, and F1-score compared to the GLM model; although the GLM model showed a slightly better precision (Table S10; supplemental material). Similar to the two previous subsystems, both models GLM and CART showed similar trends for predicted *P. vivax* malaria presence in Asia (Figs. 5 and 6). For example, the temperature in the quarter prior to the *P. vivax* malaria survey study at which malaria presence is predicted to meet maximal is 24 ^°^C (GLM model) and 24.4 ^°^C (CART model) and the thermal range where malaria presence is non-negligible, for both models is between approximately 3 ^°^C and 35 ^°^C (Fig. 5A). In contrast, precipitation of the prior quarter showed slightly different peaks in the unimodal relationship with *P. vivax* malaria, peaking at 150 mm and 200 mm of precipitation for the GLM and CART models respectively (Fig. 5B). The normalized difference vegetation index (NDVI) is one of the only predicted relationships that is not fully or mostly unimodal in both models. In the GLM model, the relationship between *P. vivax* malaria presence and NDVI seems to be monotonically increasing, although not with a constant slope, whereas the CART model exhibits a unimodal pattern with a peak at *∼*0.5 (Fig. 5D). The precipitation of the wettest quarter (BIO 16) is also different between the two models, with the GLM fit exhibiting a unimodal relationship, but the CART model predicting a similar initial peak, but higher malaria at the wettest locations (Fig. 5F).

In Fig. 6, the relationship between socio-demographic and epidemiological factors with *P. vivax* malaria in Asia are shown. Trends and values at which *P. vivax malaria* meet maximal values are very similar between the two modeling approaches. For example, high *P. vivax* malaria occurs before 2000, then both GLM and CART models showed a constant decline after 2001 (Fig. 6A). Gross domestic product per capita (GDPPC) showed a unimodal response where *P. vivax* malaria peaks around $3,294.5/year (Fig. 6B). Population density showed a positive relationship with *P. vivax* malaria which increases as population density increases (Fig. 6C). Interestingly, the marginal relationship between the relative temperature dependent basic reproductive number (*R*_0_) and *P. vivax* malaria shows a relationship that is flat or ambiguous at low values, but then increases as *R*_0_ increases towards one (Fig. 6E). The pruned tree for *P. vivax* malaria in Asia from the CART model is given in Supplemental Material (See section A.4), along with the coefficients, standard errors, and P-values from the GLM model (Table S3), a graph with the magnitude and uncertainty of the effects of each variable of the GLM model (Figure S11), and the graphs for the randomized quantile residuals and the density plot from the GLM Model (Figures S12-E and S12-F). Furthermore, in Figure S8 we are showing the correlation between variables included in this study.

## Discussion

Assessing the long-term effects of environmental, geographic, socio-demographic, and epidemiological factors on malaria is essential for public health planning, risk mitigation, and vector control, especially in the context of malaria resurgence^4,69–71^. This study examined spatially and temporally resolved predictors on *Plasmodium falciparum* and *Plasmodium vivax* malaria presence for a period of 27 years (1990–2017) at the community level for two continents, Africa and Asia. We applied two common methods to analyze binary (presence/absence) data, GLMs and CART^72–75^, and examined the similarities and dissimilarities in the prediction of malaria. Predicted malaria and its marginal association with the suite of factors showed similar responses with both approaches, but the CART model had better out of sample performance in terms of accuracy, precision, and F1-scores. Other studies have found similar results when comparing GLM and CART models^76^. Three key features of the models and data may have impacted our discussion and interpretation of results. First, because the data are aggregated from multiple studies, often focused on finding malaria, rather than designed to estimate underlying prevalence within either continent, we would expect that overall incidence is likely biased upward in this sample. Second, adjustments to spatial and temporal resolution could have introduced some distortion to the results, however we assumed these distortions are very small since the model results are very similar to other studies or are in the range of expected values. Third, all results are conditional on the inclusion of other aspects of the model, and many factors covary. Thus, we focus primarily on qualitative patterns (shapes, relative factors, etc.) rather than quantitative predictions such as specific prevalence estimates in a region.

### The role of temperature

A variety of previous work in the thermal biology of vector-borne diseases has posited that we expect to see unimodal relationships between temperature and transmission^14,77^. In our study here, across both types of fitting approaches, and a variety of temperature metrics, we found that this general pattern held, even with the presence of covarying factors, such as temperature and elevation. For example, the relationship between average temperature of the prior quarter and *P. falciparum* malaria presence in Africa was observed to be unimodal, with a predicted optimum temperature for malaria presence of 24.9 ^°^C (GLM model) and 24.8 ^°^C (CART model), with lower and upper thermal limits of 12 ^°^C and 36 ^°^C respectively. Similarly, the optimum temperature two quarters before the time the survey study took place was also hump shaped and the estimated optimum temperatures were 25.4 ^°^C (GLM model) and 25.1 ^°^C (CART model) for *P. falciparum* malaria in Africa. These temperatures are very similar to published optimum temperatures for the transmission of *P. falciparum* by *Anopheles gambiae* (25 ^°^C) and by *An. stephensi* (24.8 ^°^C)^11,15^. The native *An. gambiae mosquito* is the main malaria vector in Africa^78^ and *An. stephensi* is a recent invasive species in Africa^25^ with a greater thermal range than the native vector^15^, so we may find that these predictions would shift in the future as *An. stephensi* becomes more established.

In the models for data from Asia, similar unimodal patterns between temperature variables and malaria presence were observed. The relationship between average temperature of the 1st quarter prior to the studies showed a unimodal response for *P. vivax* malaria, with a predicted optimum temperature of 24.7 ^°^C (CART model) and 24 ^°^C (GLM model). These temperatures are slightly lower than the optimum transmission temperature suggested in Villena et al.^15^ which is 25 ^*°*^C, and lower than the optimum in Africa for the same period. This could be possible because in Asia, malaria is transmitted by multiple vectors (e.g., *An. dirus*, *An. culicifacies*, *An. maculatus*) whose thermal performance curves could be different than *An. stephensi*^79^, while in Africa *An. gambiae* is the main vector^4^. The optimum temperature two quarters prior to the survey study for *P. falciparum* and *P. vivax* malaria in Asia was in average 4 ^°^C lower than these other studies. These last results highlight the potential differences between mechanistically driven models, and correlational models (such as the ones we explore here). Although the correlational study can also capture other factors that might be related to the presence of malaria besides temperature, a signal may not be as clear especially in the presence of correlated predictors. Similarly, the mechanistic approaches could over-simplify by examining only one or a few vector species, for example.

The response of life history traits (e.g., mosquito and parasite development rate) to different constant temperatures under laboratory conditions were used in Villena et al. and Mordecai et al.^11,15^ to estimate the optimum temperature for malaria transmission. However, climatic factors do not typically have instantaneous effects on transmission; rather they may have delayed effects^80–82^. In contrast, in the field, environmental temperatures that influence parasite and vector development are rarely constant. Here we included isothermality as a factor to try to capture the separate relationship of this variability. In the fitted models here, we found that the marginal relationship of *P. falciparum* malaria in Africa with isothermality increased when temperature oscillations were up to 58%, but did not increase substantially for higher levels of variation. In Asia, both *P. falciparum* and *P. vivax* malaria showed a bell-shaped response with isothermality, highest malaria presence at oscillations around 62%. It is known that daily temperature fluctuations affect vector biology, as well as parasite development and infection rates^83,84^. Temperature oscillations also impact the abundance and age structure of *Anopheles* mosquitoes^85^. The relationships identified in this study between malaria transmission and isothermality reflect the findings of other studies. Paaijmans et al.^83^ showed that temperature fluctuations around low mean temperatures speed up biological processes, while fluctuations around high mean temperatures slow down biological processes of the vector and the parasite^84^. Zhao et al.^84^ found that large daily temperature oscillations speed up malaria incidence in cooler environmental conditions, but in warmer regions large daily temperature oscillations will slow down malaria incidence.

### The role of precipitation

In this study, the four precipitation variables (i,e., precipitation one and two quarters prior to the survey study start and precipitation of the wettest and driest quarters) showed a curve-like response with a positive relationship with *P. falciparum* malaria in Africa up to a certain level of cumulative precipitation and then a decline. For example, precipitation of the wettest quarter (BIO16) positively impacts *P. falciparum* malaria, to a peak of 1350 mm and 930 mm of quarterly cumulative rainfall for the GLM and CART models respectively; and greater amounts of rainfall have a negative effect on malaria. Similar patterns were observed with precipitation one and two quarters prior to the survey study, and for precipitation of the driest quarter (BIO17). It is well established that precipitation plays a major role in the availability of habitat for immature stages (i.e., eggs, larvae, pupae) of *Anopheles* mosquitoes, however excess precipitation can flush away immature life stages from these habitats^86^. Most of the African continent is classified as semi-arid, with an average annual precipitation of 469.9 mm in a single summer wet season (December—March), except countries located near the equator where two wet seasons occur, with increased rainfall^87^. Countries located between 10 N and 10 S latitudes have optimal amounts of precipitation (< 1350 mm) for *Anopheles* mosquito development, with the exception of small areas that experience more than 1350 mm rainfall for at least three months of the year, such as in Gambia, Guinea-Bissau, Guinea, Sierra Leone, Liberia, Nigeria, Cameroon, Equatorial Guinea, Gabon, and Madagascar^87^. Yet, outside these periods of heavy rains, these areas are also optimal for mosquito development in terms of precipitation^87^. Changes in rainfall patterns due to global climate change can have profound consequences on mosquito development and transmission of vector-borne diseases^7,88,89﻿^. Rainfall trends have already been changing, such as decreases in East Africa and increases in Southern Africa^89,90^.

In Asia, the models revealed counterintuitive relationships between *P. falciparum* malaria presence and precipitation. Increases in mean precipitation one and two quarters prior to the survey study corresponded with a constant decline in malaria. Precipitation of the driest quarter (BIO 17) also had a strong negative correlation with malaria presence, where despite a peak at 210 mm of rainfall (GLM and CART models), malaria activity sharply decreases with increased precipitation. Similar relationships were found for *Plasmodium vivax* malaria presence, wherein the precipitation of the driest quarter (BIO17), and the mean precipitation of the second quarter prior the survey study show strong negative relationships. As with *P. falciparum*, precipitation of the driest quarter showed peak *P. vivax* presence when precipitation is around 210 mm (GLM and CART models), after which malaria decreases with increased precipitation. *Plasmodium vivax* presence also increases with precipitation of the wettest quarter (BIO16) until an optimum (900 mm) is reached, after which malaria presence decreases with continued rainfall. These findings align with those presented in^91^, which showed that 60 to 80 mm of monthly rainfall is enough to increase availability of breeding sites for mosquitoes and indirectly drive malaria transmission. This echoes findings in Africa, where locally intense rain events that exceed optima can decrease early-stage larvae and pupae through the flushing of ovipositional habitats^92^.

### The role of normalized difference vegetation index – NDVI

NDVI relates to malaria in Asia with a bell-shaped response, where malaria presence of both *P. falciparum* and *P. vivax* was rise up to an optimum value (*P. falciparum*: 0.52 (GLM) and 0.54 (CART); *P. vivax*: 0.4 (GLM) and 0.5 (CART)), beyond which malaria was predicted to decrease. Multiple studies have shown higher numbers of malaria cases in areas with low to medium density of vegetation. For example, the number of malaria cases increases when forested areas are deforested, which results in NDVI values similar to those that favor malaria presence in this study^93^. The study of Nihei et al.^94^ also found a positive correlation with *P. falciparum* malaria when NDVI values are of 0.4 + for at least 6 months. The GLM model for *P. vivax* showed a constant increase of malaria as NDVI increases up to 0.8 index; however, between 0.4 and 0.8 the rate of increase is minimal.

The NDVI also showed a bell-shaped response with *P. falciparum* malaria which was positively impacted up to an optimum value of NDVI = 0.73, beyond which malaria decreased. However, this variable is not determinant for *P. falciparum* malaria presence in Africa. This could be because of the difference in vegetation cover and its variation from country to country in Africa. For example, In Uganda, malaria incidence was greater when the average NDVI = 0.72^95^ while in Kenya the overall effect of NDVI was highest when NDVI was below 0.4^96^.

### The role of socio-economic and demographic factors

Gross domestic product per capita (GDPPC) is associated with increased *P. falciparum* in Africa, and both *P. falciparum* and *P. vivax* in Asia. Malaria increases with GDPPC to a certain point, after which it decreases. Notably, in Asia most countries have a GDPPC greater than the thresholds identified in this study ($2,322 for *P. falciparum*; $3,294.5 for *P. vivax*). In contrast, many countries in Africa have a GDPPC well below the regional threshold ($2,250) for high malaria prevalence^97^. Multiple studies have shown a strong correlation between high malaria burden and low GDPPC^98^, or low per capita income^97,99^. For example, the Sarma et al. study^99^ found a 10% decrease in malaria incidence with an increase of 0.3% in average income per capita. This is important, as malaria endemic countries also show some of the lowest rates of economic growth globally^100^. This could be worse in coming years if we consider that current and projected annual gross domestic product growth is smaller, particularly for regions in Africa^100,101^.

Population density had a weakly positive relationship with *P. falciparum* malaria in Africa. Increase in population density was associated with increasing malaria until a density of about 898 people per square kilometer; then malaria decreased as population density increased. Multiple studies have found similar results where malaria transmission increases as population density increases, up to a peak of around 1000 pp/km^2^, and then malaria transmission decreases in populated areas with densities greater than 1000 pp/km^2^^20^. Malaria has been considered as less of a problem in urban areas compared to rural areas in Africa^102^. However, the relationship between population density and malaria burden may drastically change in the future if the invasive mosquito *An. stephensi* became established in Africa, due to its proclivity for reproducing in urban and highly populated areas^25,103^. In southeast Asia, malaria is also more of a problem in rural areas than in very dense cities^104^. Economic development and environmental changes (e.g. drainage of mosquito breeding sites, improved housing) during the twentieth century have reduced the incidence of malaria in urban contexts^105^.

### Other factors driving malaria

The year that malaria survey studies took place and the human development index showed a strong and weak negative relationship with *P. falciparum* malaria respectively. Both the GLM and the CART model showed a sharp decline in malaria from 2000 to 2010. However, after 2010, the GLM model showed a slowdown in the decline of *P. falciparum* malaria while the CART model showed a constant increase of *P. falciparum* malaria. We need to take into account that our *P. falciparum* malaria survey data comes mostly from the east part of Africa (Ethiopia, Somalia, Kenya, Tanzania, Mozambique, Zimbabwe, and Madagascar) where presence of malaria is higher and less from the west coast of Africa (The Gambia, Guinea-Bissau, Nigeria, Cameroon, and Sao Tome and Principe). Our models capture well the start of the malaria decline which started around 2000 when there was increased funding for malaria control^106^. Also, our models capture the resurgence of malaria in the last 8 to 10 years. The last malaria report from the World Health Organization (WHO) showed that the number of malaria cases and deaths had been increasing globally since 2016^4^ and in Africa since 2014^4,107^. Multiple studies found that the resurgence of malaria in Africa is related to multiple factors such as the rebound or delayed malaria^108^, insecticide resistance or ineffective new insecticides^109,110^, the effects of climate change^15^, and the presence of a new vector, the invasive *An. stephensi*^103^. Both of our models showed that *P. falciparum* malaria is higher in countries with low human development index (HDI), a summary of health, education, and income indicators, and malaria decreases as the HDI increases. Multiple studies had found similar results where malaria incidence rates are higher in countries with lower values of HDI^111^.

Similarly to the results for the African continent, both the GLM and CART models captured the start of *P. falciparum* malaria decline in the 2000s in Asia well. Both of the models captured a slowing down in the decline of malaria around 2010 and a resurgence following this, especially the GLM model. The last malaria report from the World Health Organization (WHO) showed that the number of malaria cases and deaths had been increasing globally since 2016^4^. Similarly to Africa, the causes of malaria resurgence are multiple such as insecticide resistance or ineffective new insecticides^109,110^ and the effects of climate change^15^. Both models showed a gentle decrease of *P. vivax* malaria from 1990 to 2003, followed by a rapid decrease of *P. vivax* malaria after 2003. Finally, *P. falciparum* malaria in Africa was positively correlated with the basic reproductive number (*R*_0_). The basic reproductive number is an important and widely used indicator of the dynamics of malaria^112,113^.

## Conclusions

This study presents important progress in understanding the long-term influence of environmental, geographic, socio-demographic, and epidemiological factors on malaria in Africa and Asia. This provides key information on the relative roles of climate driver timing, key demographic features of affected populations, and geographies, that can inform planning strategies and interventions to reduce malaria burden in these two continents. While a ’one model fits all’ approach may not be globally appropriate for predictive frameworks, a CART framing allows us to see how continental differences in responses to this suite of variables arise, and captures the changing dynamics of malaria throughout the time-frame of the data.

### Supplementary Information

Supplementary Information 1.

## Data Availability

Malaria survey data was collected by the Malaria Atlas Project following the General Data Protection Regulation (GDPR) and associated data protection legislation, https://malariaatlas.org/privacy-policy/. Malaria survey data used in this study is publicly available online (https://malariaatlas.org/) as well as all environmental, geographic, and socio-demographic raster layers used in this study as described within the paper. R Code for creating model outputs is available publicly on Zenodo at 10.5281/zenodo.11194470.

## List of symbols

GLM: Generalized linear models
CART: Classification and regression trees
GDPPC: Gross domestic product per capita
HDI: Human development index
MBDs: Mosquito-borne diseases
NDVI: Normalized difference vegetation index
R_*0*_: Basic reproductive number
USGS: United States geological survey
FEWS-NET: Famine early warning systems network
MODIS: Moderate resolution imaging spectroradiometer
NASA: National aeronautics and space administration
NetCDF: Network common data form
GRUMP: Global Rural–Urban Mapping Project
MAP: Malaria Atlas Project
RQRs: Randomized quantile residuals
