# Supplementary material for: Influence of environmental, geographic, socio-demographic, and epidemiological factors on presence of malaria at the community level in two continents
Source: Sci Rep. 2024 Jul 20;14:16734. doi: 10.1038/s41598-024-67452-5 (PMC11271557; doi:10.1038/s41598-024-67452-5)
Supplement: Supplementary file 1 — Supplementary Information 1. [file 41598_2024_67452_MOESM1_ESM.docx]

**Supplementary Information.** Villena, O.C., A. Arab, C.A. Lippi, S.J. Ryan, and L.R. Johnson. Influence of environmental, geographic, socio-demographic, and epidemiological factors on presence of malaria at the community level in two continents. Scientific Reports

**Appendix 1:** Supplementary Information

# Supplemental Material: Figures

**
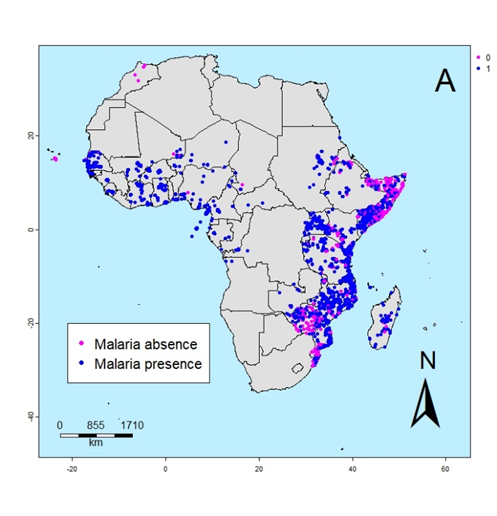

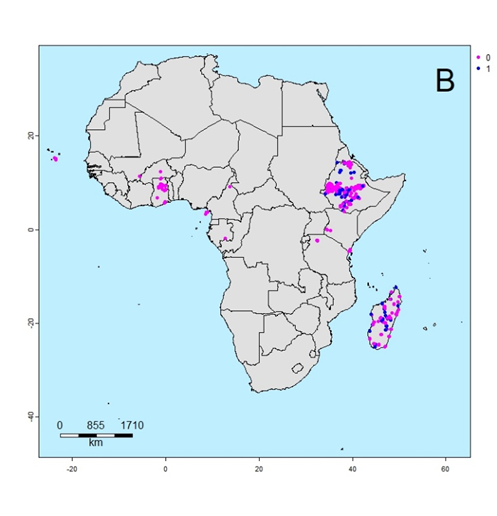
**

**
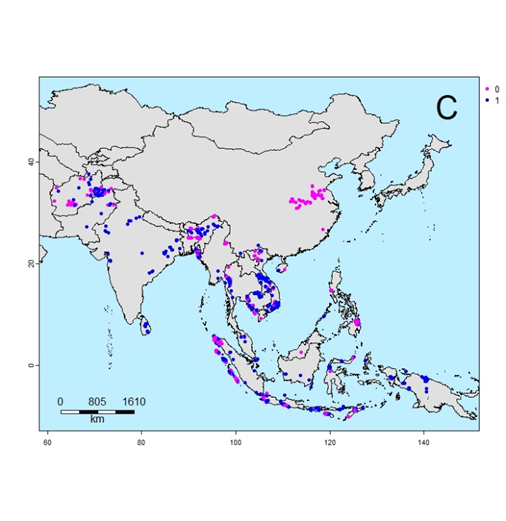

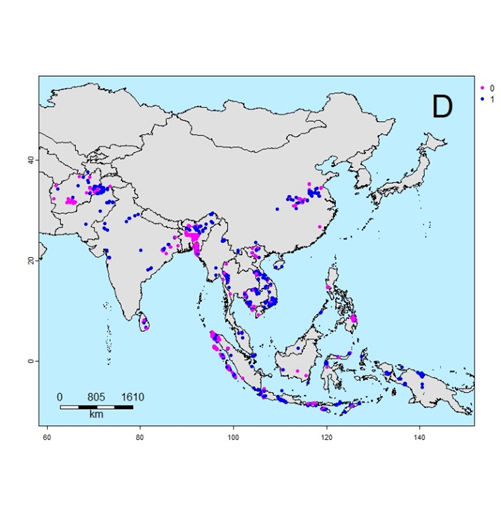
**

Figure S1: Malaria absence/presence at the community level from survey data in space for: A) *P. falciparum* in Africa, B) *P. vivax* in Africa, C) *P. falciparum* in Asia, and D) *P. vivax* in Asia.

## Marginal predictions for temperature, precipitation, and the basic reproductive number (*R*_0_) two quarters prior to the start of the malaria survey for *P. falciparum* and *P. vivax* malaria in Africa and Asia


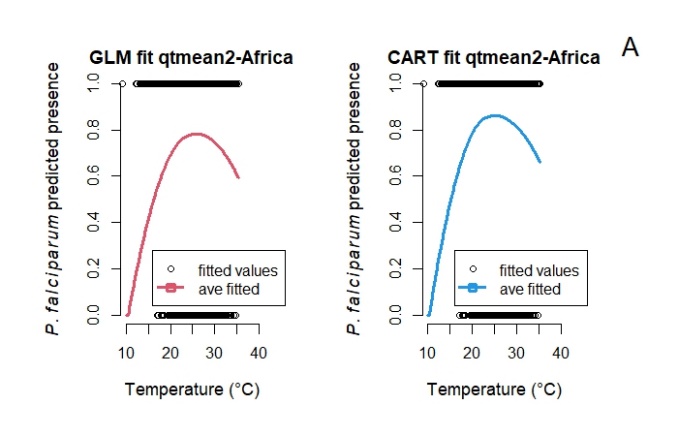

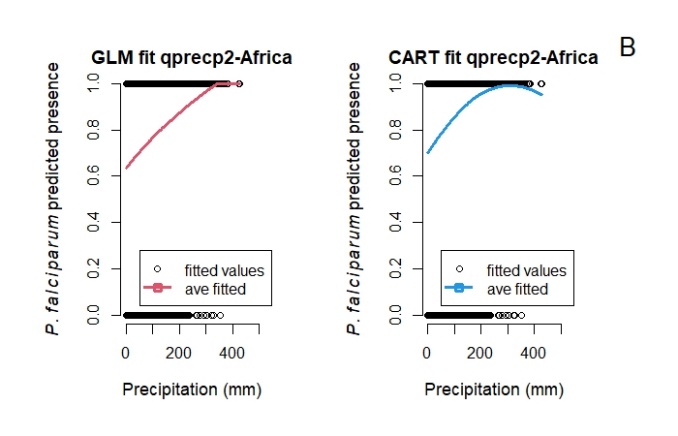


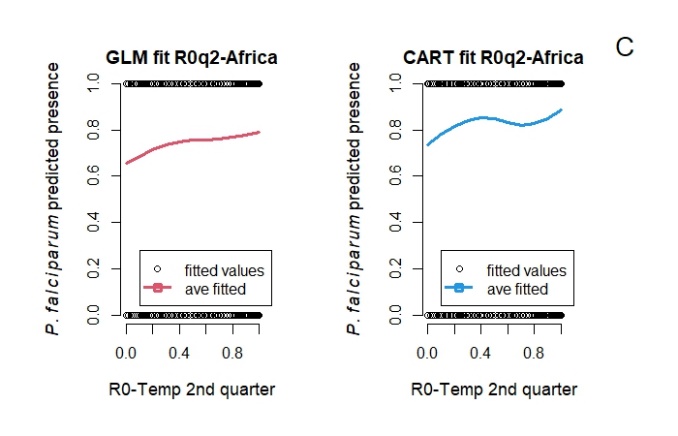


Figure S2: Marginal predictions based on particular predictors A) Temperature 2nd quarter prior to the start of the survey study B) Precipitation 2nd quarter prior to the start of the survey study, and C) The basic reproductive number (*R*0) 2nd quarter prior to the start of the survey study for *P. falciparum* malaria in Africa.


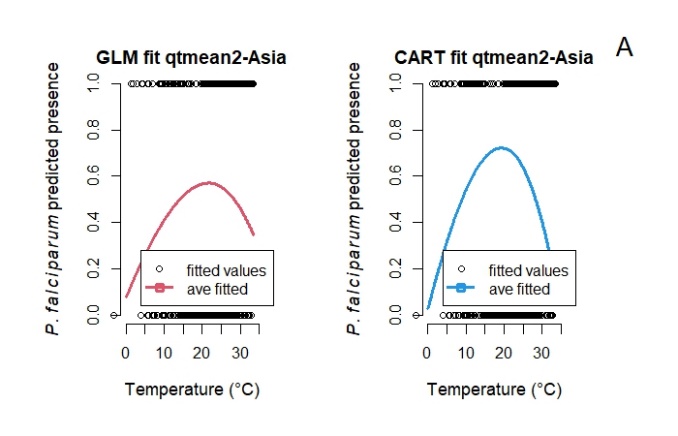

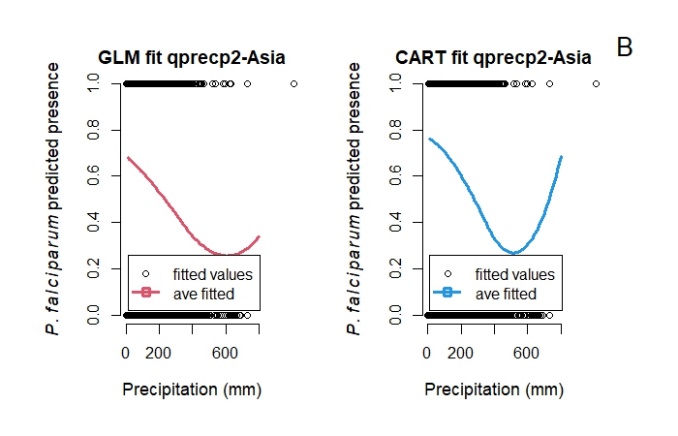


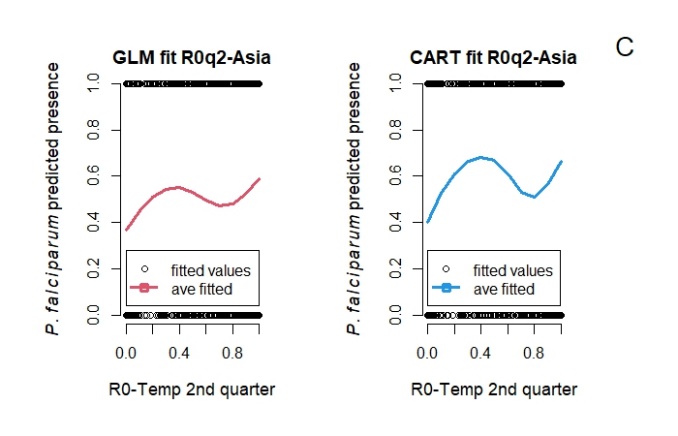


Figure S3: Marginal predictions based on particular predictors A) Temperature 2nd quarter prior to the start of the survey study B) Precipitation 2nd quarter prior to the start of the survey study, and C) The basic reproductive number (*R*0) 2nd quarter prior to the start of the survey study for *P. falciparum* malaria in Asia.


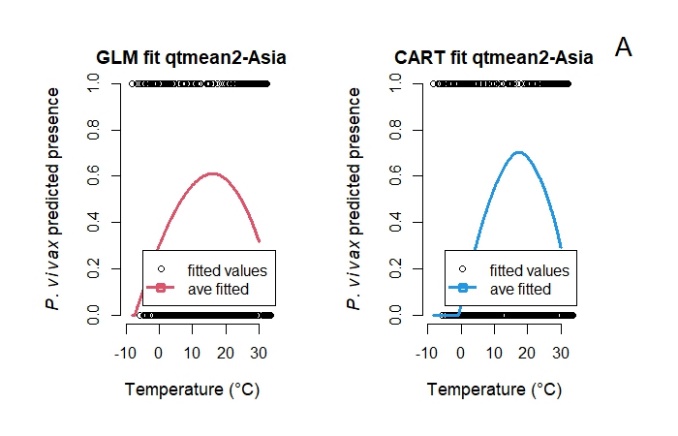

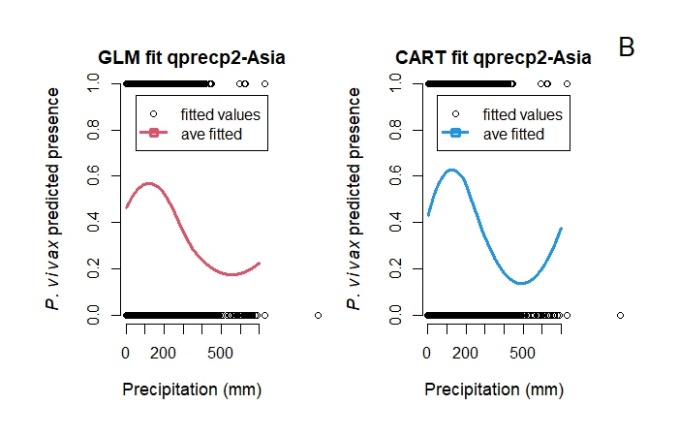


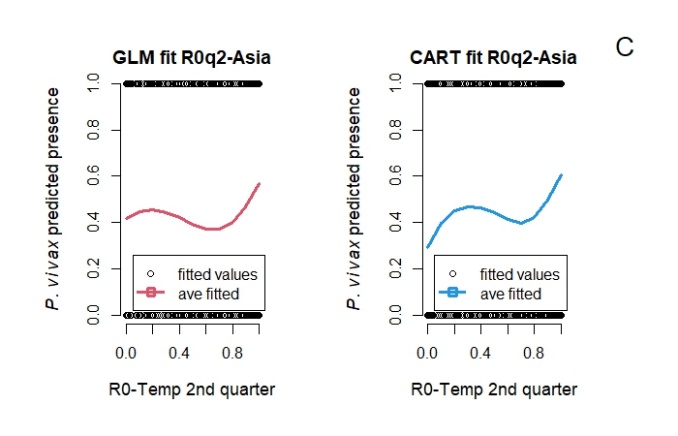


Figure S4: Marginal predictions based on particular predictors A) Temperature 2nd quarter prior to the start of the survey study B) Precipitation 2nd quarter prior to the start of the survey study, and C) The basic reproductive number (*R*0) 2nd quarter prior to the start of the survey study for *P. vivax* malaria in Asia.

## CART analysis: Pruned tree for *P. falciparum* malaria presence in Africa

In Figure S5, we show the pruned tree from the CART analysis for *P. falciparum* malaria presence in Africa. The tree shows that 12 of the 15 predictors are considered to be predictive of *P. falciparum* malaria presence – only NDVI, population density, *R*_0_ from two previous quarters were entirely excluded from the pruned tree. The rest of the variables in the tree appear in (sometimes) surprising combinations to group subsets of the data, suggesting possible interactions that predict malaria presence. We observed that the precipitation of the wettest quarter (BIO 16) is the most important determinant of *P. falciparum* malaria presence in Africa, as the initial branching depends on this variable (bio16 *<* 387 mm). At the second level, we observed that the year in which the survey study took place and elevation (left and right child nodes respectively) are correlated with *P. falciparum* malaria presence. The left child node has a value of survey study start year *≥* 2009 and the right child node has a value of elevation *≥* 1,665 m. At the next level, in the left child node, average temperature of second previous quarter (qtmean *<* 24) and the precipitation of driest quarter (bio17 *<* 19) are important variables, and in the right child node the survey study start year (year start *≥* 2006) and HDI (hindex *≥* 0.5) are important variables.

At the bottom of the tree, each leaf in the tree diagram shows the probabilities of *P. falciparum* malaria presence. For example, the values at the left-most leaf in Figure S5 indicate that the probability of *P. falciparum* malaria absence (i.e., 0) is 91% if the precipitation of the wettest quarter is *<* 387 mm, the year of the survey study was 2009 or above, and the average temperature two quarters prior the survey study was *<* 24*^◦^*C. The values at the right-most leaf in Figure S5 indicate that the probability of *P. falciparum* malaria presence (i.e., 1) is 9% if precipitation of the wettest quarter is *≥* 387 mm, the elevation is *<* 1,665, and the human development index is *<* 0.5.

## CART analysis: Pruned tree for *P. falciparum* malaria presence in Asia

From the CART analysis, we show the pruned tree for *P. falciparum* malaria presence in Asia (Figure S6). Of the 15 variables used in the model (Table 2), 6 variables are considered to have an effect on the prediction of *P. falciparum* malaria presence in Asia: the year at which the malaria survey study took place, isothermality (BIO 3), the human development index, precipitation of driest quarter, average temperature one quarter prior to the survey study month, and the normalized difference vegetation index (NDVI) (Figure S6).

The variable that best split this data set is the year at which the malaria survey study took place (year start *≥* 2006). The second most important determinants of *P. falciparum* malaria presence are isothermality (bio3 *<* 46) at the left child node, and the human development index (hindex *<* 0.38) at the right child node. At the third level, the most important determinants are human development index (hindex *≥* 0.45 and 0.65) at the left child nodes and precipitation of the driest quarter (BIO 17 *≥* 542) and isothermality (BIO 3 *<* 33) at the right child nodes. We continue in a similar way in the next levels until branches terminate in leaves (Figure S6).

Each leaf in the tree diagram shows the probabilities of *P. falciparum* malaria presence in Asia. For example, the values at the left-most leaf in (Figure S6) indicate an 88% probability of absence of *P. falciparum* malaria and 12% probability of presence of *P. falciparum* malaria in Asia if the year at which the malaria survey study took place is *≥* 2006, isothermality is *<* 46, and human development index *≥* 0.43.


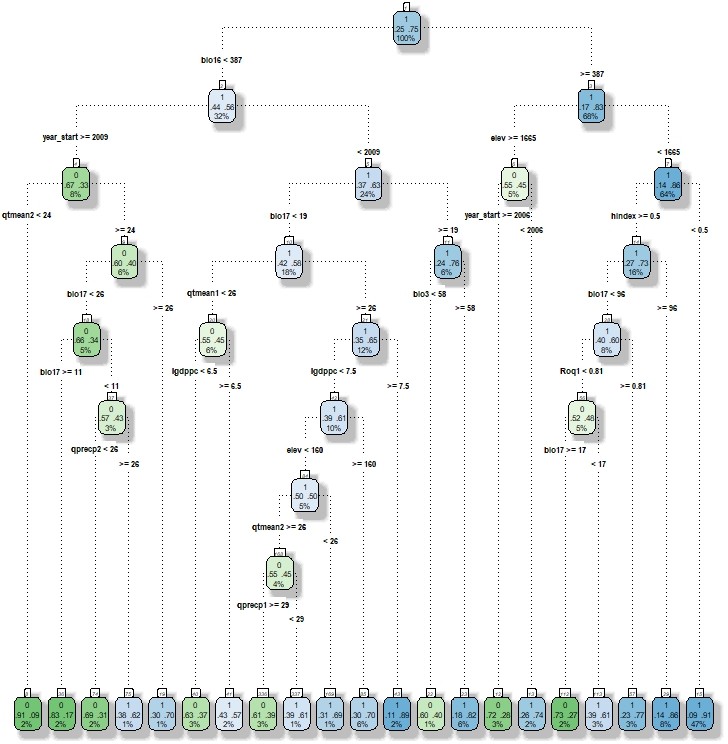


Figure S5: Pruned tree showing the parameters and values that best predict *P. falciparum* malaria presence in Africa. The splits from each node follow the rule left=YES. Each node provides the following information: the predicted class (absence (0) or presence (1)), the probability of presence/absence which adds to 1, and the percentage of observations in the node. The color scale shows the presence (blue) to absence (green) of *P. falciparum* malaria, color changes towards white as malaria presence/absence approaches 50/50 percent. Bio3: isothermality, bio16: precipitation of wettest quarter, bio17: precipitation of driest quarter, elev: elevation (m), year start: year at which the survey study took place, qtmean1 and qtmean2: average temperature one and two quarters prior to the survey study time, qprecp1 and qprecp2: average precipitation one and two quarters prior to the survey study time, hindex: human development index, lgdppc: log gross domestic product per capita, and *R*0q1: basic reproductive number first quarter.


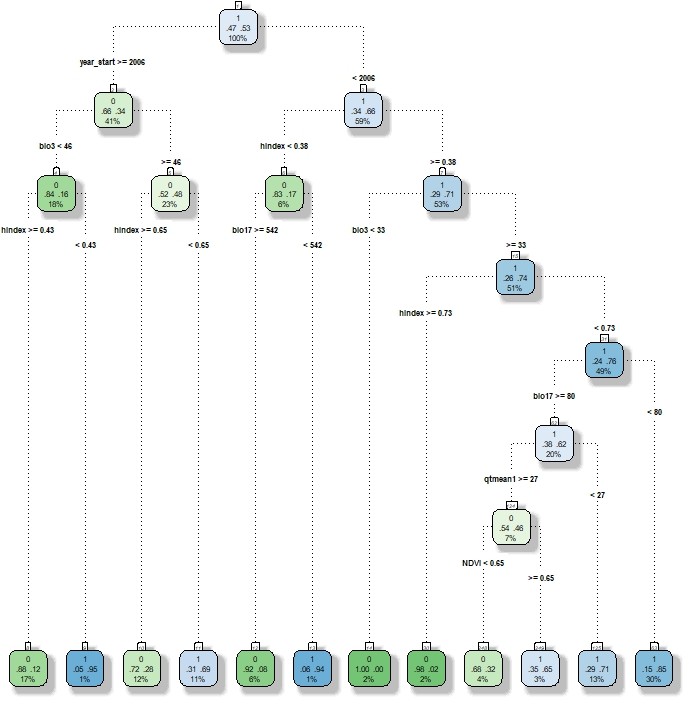


Figure S6: Pruned tree showing the parameters and values that most explain *P. falciparum* malaria presence in Asia. The splits from each node follow the rule left=YES. year start: year at which the survey study took place, bio3: isothermality (%), hindex: the human development index, bio17: precipitation of driest quarter, qtmean1: average temperature one quarter prior to the survey study time, NDVI: the normalized difference vegetation index.

## CART analysis: Pruned tree for *P. vivax* malaria presence in Asia

From the CART analysis, we show the pruned tree for the full data set for *P. vivax* malaria presence in Asia (Figure S7). In the final model, 8 variables are considered to have an effect on the prediction of *P. vivax* malaria presence in Asia, with the exception of isothermality, mean temperature of the 2nd quarter prior to the survey study date, mean precipitation of the 1st quarter prior to the survey study date, the basic reproductive number (*R*_0_) for the 1st quarter, elevation, population density, and NDVI (Figure S7).

The variable that best splits this dataset is the year in which the survey study took place. This is the most important determinant of *P. vivax* malaria presence, as the initial branching depends on this variable (survey study start year *≥* 2005). The second most important determinants of *P. vivax* malaria presence are the GDPPC in the left child node (lGDP *<* 9.1) and the precipitation of the driest quarter in the right child node (bio17 *≥* 636). At the next level, the precipitation of the wettest quarter (bio16 *≥* 1221) in the left child node and GDPPC (lGDP *<* 7.7) and the human development index (hindex *≥* 0.73) in the right child node are the most important.

Each leaf in the tree diagram is labeled with the probability that the response variable (*P. vivax* malaria presence) is true. For example, the values at the left-most leaf in Figure S7 indicate that the probability of *P. vivax* malaria absence is 91% if the year at which the survey study took place is *≥* 2005, log(gdppc) *<* 9.1, and precipitation of the wettest quarter is *≥* 1221 mm (Figure S7).


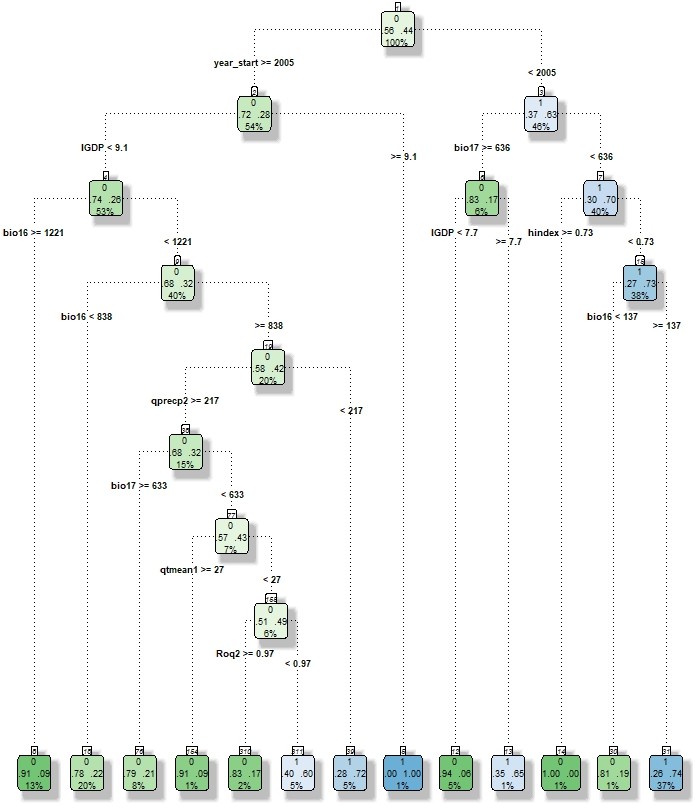


Figure S7: Pruned tree showing the parameters and values that most explain *P. vivax* malaria presence in Asia. The splits from each node follow the rule left=YES. year start: year at which the survey study took place, lGDPPC: log of the gross domestic product per capita, bio17: precipitation of the driest quarter, bio16: precipitation of the wettest quarter, hindex: the human development index, qprecp2: average precipitation two quarters prior to the survey study time, qtmean1: average temperature one quarter prior to the study time, Roq2: the basic reproductive number two quarters prior to the survey study time.

## Correlation between variables included in the study.


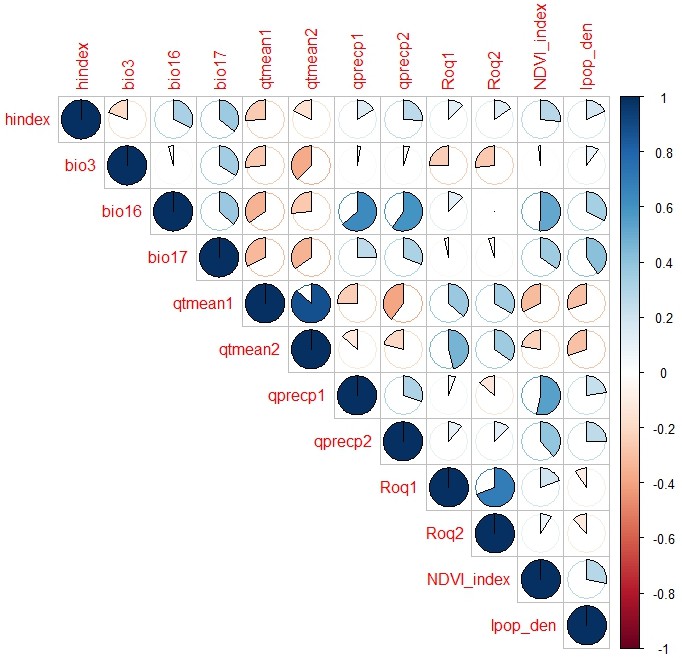


Figure S8: Pearson correlation coefficients for the variables used in this study. hindex: human development index, bio3: isothermality, bio16: precipitation of wettest quarter, bio17: precipitation of driest quarter, qtmean1: average temperature of previous quarter, qtmean2: average temperature before previous quarter, qprecip1: average precipitation of previous quarter, qprecip2: average precipitation before previous quarter, *R0*q1: basic reproductive number of the previous quarter, *R0*2: basic reproductive number before the previous quarter, NDVI index: normalized difference vegetation index, and lpop den: log of population density.

## Magnitude and uncertainty of the effects for each variable of the GLM models


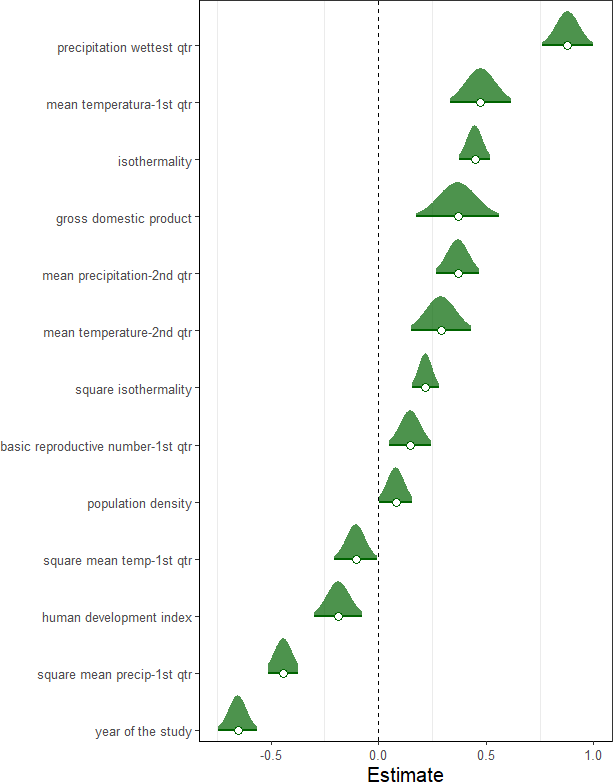


Figure S9: Magnitude and uncertainty of the effects for each variable of the GLM model for *P. falciparum* malaria presence in Africa.


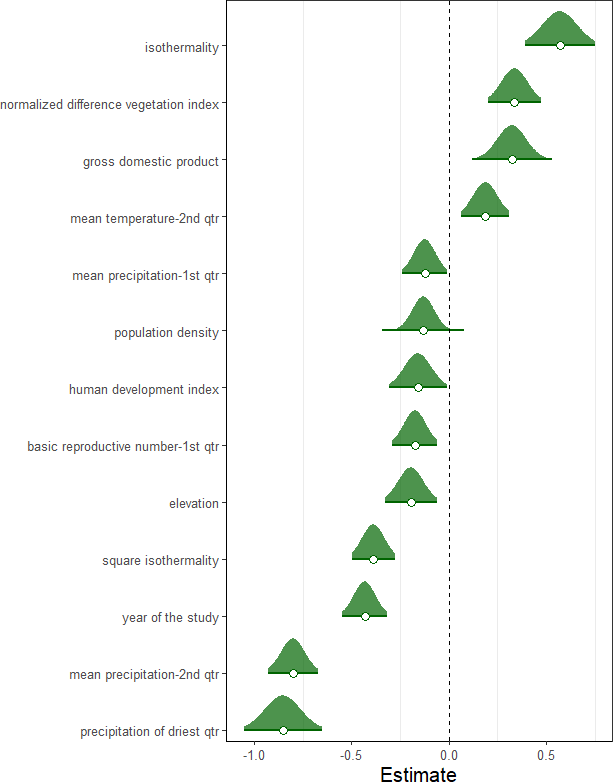


Figure S10: Magnitude and uncertainty of the effects for each variable of the GLM model on *P. falciparum* malaria presence in Asia.


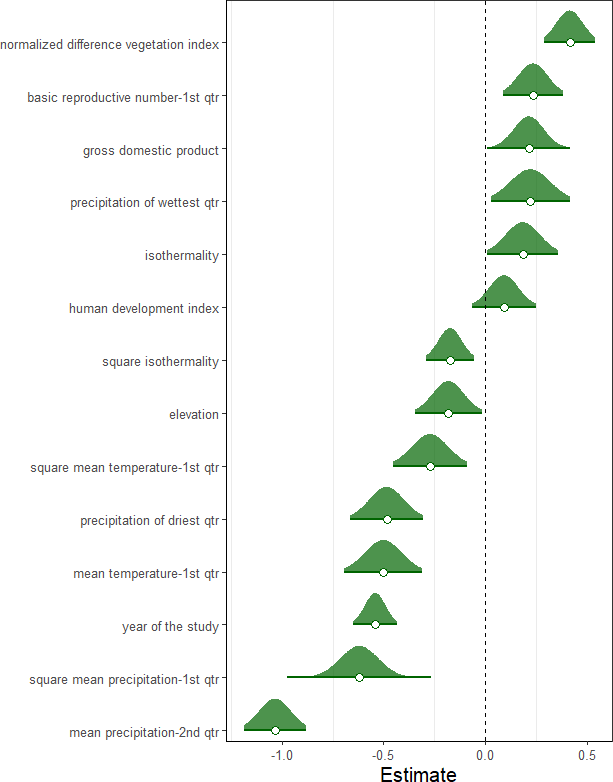


Figure S11: Magnitude and uncertainty of the effects for each variable of the GLM model on *P. vivax* malaria presence in Asia.

## Randomized quantile residuals and density plots from GLM models


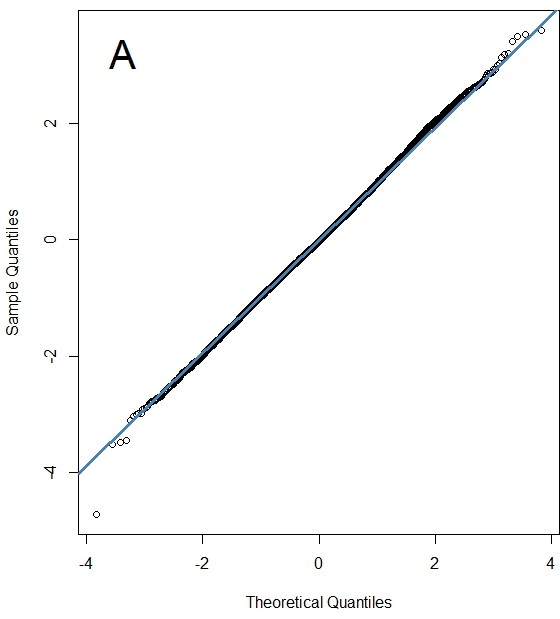

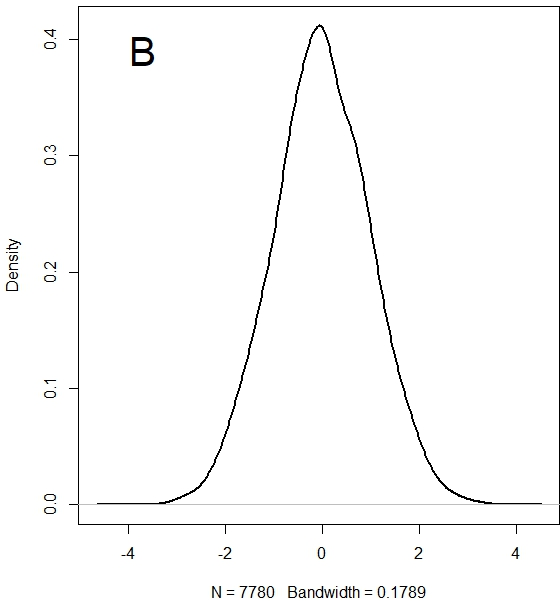

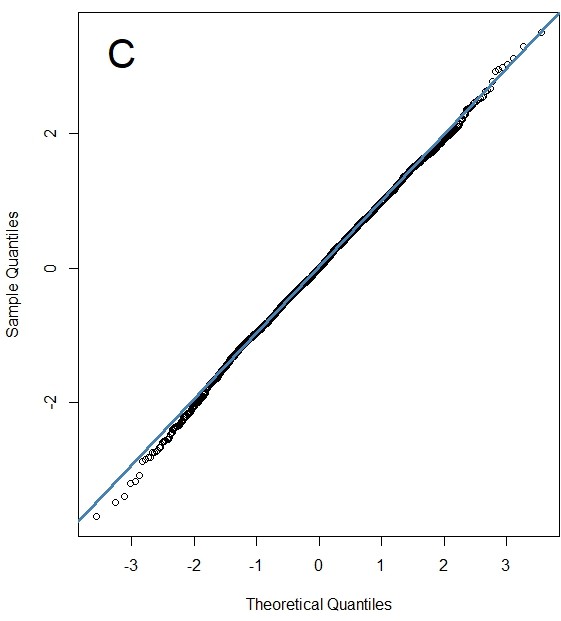

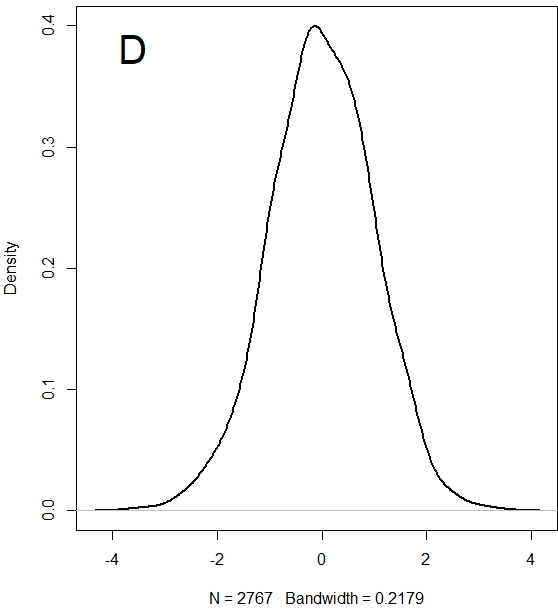

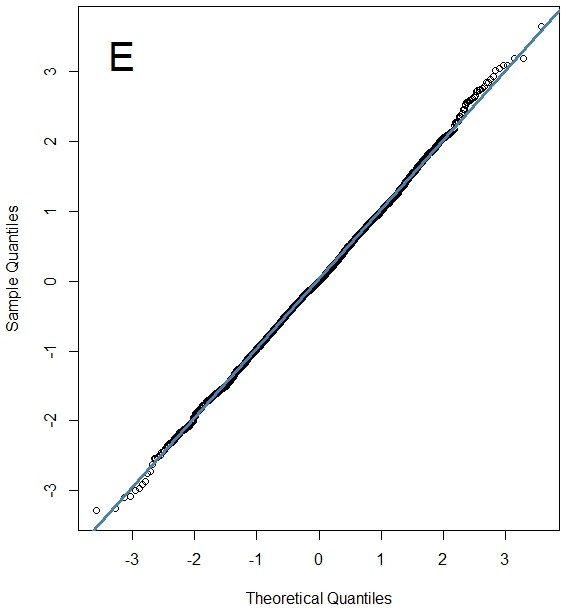

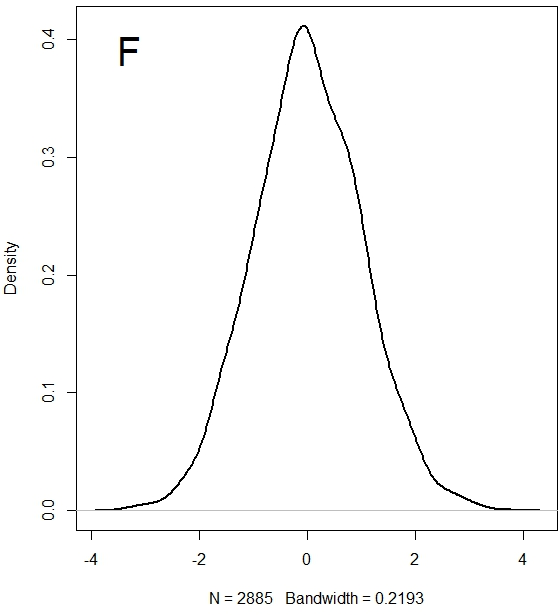


Figure S12: Q-Q plot for the quantile residuals extracted in R using *qresid* function in the package *statmode* and density plots extracted using the function *density* for the best fitted models: A) Q-Q plot for *P. falciparum* in Africa, B) density plot for *P. falciparum* in Africa, C) Q-Q plot for *P. falciparum* in Asia, D) density plot for *P. falciparum* in Asia, E) Q-Q plot for *P. vivax* in Asia, and F) density plot for *P. vivax* in Asia.

# Supplemental Material: Tables

- 1. **Summary glm models for** *P. falciparum* **and** *P. vivax* **malaria presence in Africa and Asia**

|  | Estimate | S.E. |
| --- | --- | --- |
| Intercept | 1*.*55 | (0*.*05)*^∗∗∗^* |
| precipitation of the wettest quarter | 6*.*26 | (0*.*39)*^∗∗∗^* |
| start year of the survey study | *−*211*.*42 | (13*.*34)*^∗∗∗^* |
| mean temperature - 2nd quarter | 2*.*94 | (0*.*72)*^∗∗∗^* |
| isothermality | 4*.*72 | (0*.*38)*^∗∗∗^* |
| mean precipitation - 1st quarter (sq) | *−*8*.*53 | (0*.*82)*^∗∗∗^* |
| basic reproductive number (RO) - 1st quarter | 0*.*43 | (0*.*14)*^∗∗^* |
| isothermality (sq) | 20*.*57 | (2*.*93)*^∗∗∗^* |
| mean precipitation - 2nd quarter | 2*.*29 | (0*.*32)*^∗∗∗^* |
| mean temperature - 1st quarter | 4*.*62 | (0*.*68)*^∗∗∗^* |
| gross domestic product per capita | 5*.*42 | (1*.*29)*^∗∗∗^* |
| human development index | *−*1*.*43 | (0*.*42)*^∗∗∗^* |
| mean temperature - 1st quarter (sq) | *−*7*.*55 | (3*.*22)*^∗^* |
| population density | 2*.*06 | (1*.*01)*^∗^* |

*∗∗∗p <* 0*.*001; *^∗∗^p <* 0*.*01; *^∗^p <* 0*.*05; *^·^p <* 0*.*1

Table S1: Summary of the GLM model for *P. falciparum* presence in Africa. Squared terms (sq)

|  | Estimate | S.E. |
| --- | --- | --- |
| Intercept | 0*.*69 | (0*.*09)*^∗∗∗^* |
| isothermality (sq) | *−*12*.*89 | (1*.*95)*^∗∗∗^* |
| mean precipitation - 2nd quarter | *−*5*.*32 | (0*.*41)*^∗∗∗^* |
| start year of the survey study | *−*163*.*94 | (21*.*16)*^∗∗∗^* |
| isothermality | 2*.*78 | (0*.*44)*^∗∗∗^* |
| precipitation of the driest quarter | *−*3*.*44 | (0*.*37)*^∗∗∗^* |
| elevation | *−*1*.*92 | (0*.*64)*^∗∗^* |
| normalized difference vegetation index | 1*.*50 | (0*.*31)*^∗∗∗^* |
| gross domestic product per capita | 2*.*50 | (0*.*58)*^∗∗∗^* |
| human development index | *−*1*.*58 | (0*.*68)*^∗^* |
| mean precipitation - 1st quarter (sq) | *−*0*.*69 | (0*.*31)*^∗^* |
| population density | *−*3*.*08 | (1*.*36)*^∗^* |
| basic reproductive number (RO)- 1st qtr | *−*0*.*84 | (0*.*28)*^∗∗^* |
| mean temperature - 2nd quarter | 1*.*64 | (0*.*56)*^∗∗^* |

*∗∗∗p <* 0*.*001; *^∗∗^p <* 0*.*01; *^∗^p <* 0*.*05; *^·^p <* 0*.*1

Table S2: Summary of the GLM model for *P. falciparum* presence in Asia. Squared terms (sq)

|  | Estimate | S.E. |
| --- | --- | --- |
| Intercept | 0*.*43 | (0*.*11)*^∗∗∗^* |
| start year of the survey study | *−*213*.*21 | (20*.*16)*^∗∗∗^* |
| mean precipitation - 2nd qtr | *−*6*.*89 | (0*.*54)*^∗∗∗^* |
| human development index | 0*.*68 | (0*.*55) |
| normalized difference vegetation index | 1*.*63 | (0*.*26)*^∗∗∗^* |
| isothermality (sq) | *−*5*.*75 | (1*.*96)*^∗∗^* |
| mean precipitation - 1st quarter (sq) | *−*10*.*09 | (1*.*55)*^∗∗∗^* |
| precipitation of the driest quarter | *−*1*.*97 | (0*.*35)*^∗∗∗^* |
| basic reproductive number (RO)- 1st qtr | 0*.*79 | (0*.*25)*^∗∗^* |
| mean temperature - 1st qtr | *−*3*.*65 | (0*.*66)*^∗∗∗^* |
| square mean temperature - 1st qtr | *−*6*.*05 | (2*.*01)*^∗∗^* |
| elevation | *−*1*.*46 | (0*.*62)*^∗^* |
| gross domestic product per capita | 1*.*67 | (0*.*58)*^∗∗^* |
| precipitation of the wettest quarter | 1*.*26 | (0*.*57)*^∗^* |
| isothermality | 0*.*84 | (0*.*39)*^∗^* |

*∗∗∗p <* 0*.*001; *^∗∗^p <* 0*.*01; *^∗^p <* 0*.*05; *^·^p <* 0*.*1

Table S3: Summary of the GLM model for *P. vivax* presence in Asia. Squared terms (sq)

- - 1. **Model performance assessment: CART and GLM models**

We assessed the performance of the CART and GLM models for *P. falciparum* malaria presence in Africa and *P. vivax* in Asia which are the two richest data sets in our study (Table 1). To assess the prediction performance, we use a validation data set (30% of the total amount of data) to estimate the classification ability of these two models. We use four different metrics: accuracy, precision, recall, and the F1 score based on each model’s confusion matrix [1, 2].

The confusion matrix is a table that summarizes how successful classification models are at predicting samples belonging to various classes as summarized at Table S4. To build the confusion matrix, a set of predictions are calculated based on the model so that they can be compared to the actual targets. To do this, we used the function *predict* in the software R, which computes the predictions on the validation data set (0.3 of each data set). In the confusion matrix, each row represents a calculated, also called actual, target, while each column represents a predicted target where it is count the number of times “TRUE” instances are classified either as “TRUE” or “FALSE” following the next design. (Table S4).

Predicted

FALSE TRUE

Calculated FALSE True Negative (TN) False Positive (FP) TRUE False Negative (FN) True Positive (TP)

Table S4: Confusion matrix

Using the information of the confusion matrix, we estimated the model accuracy. Model ac- curacy is defined as the total correctly classified samples divided by the total number of classified samples. The accuracy equation in terms of the confusion matrix is express as:

*Accuracy* =

*TP* + *TN*

*TP* + *TN* + *FP* + *FN*

(1)

The model precision, also called positive predictive value, refers to the accuracy of the positive predictions (False and True positive). Precision is calculated using the following equation:

*Precision* =

*TP TP* + *FP*

(2)

The model recall, also called sensitivity, is the ratio of positive instances that are correctly detected by the classifier, and it is calculated using the following equation:

*Recall* =

*TP TP* + *FN*

(3)

The F1-score is based on precision and recall. The F1-score is a weighted mean of these two metrics, meaning it gives more weight to the lower values and it is calculated using the following equation:

$F1-score=2*\frac{Precision*Recall}{Precision+Recall}$ (4)

**Model performance assessment for** *Plasmodium falciparum* **malaria presence in Africa.**

We built confusion matrices for the CART (Table S5) and GLM (Table S6) models. The first row of the CART confusion matrix shows the absence (0) of *P. falciparum* malaria. Of the 585 cases, 198 were correctly classified as absence of *P. falciparum* malaria, while the remaining (387) were wrongly classified as presence (1) of *P. falciparum* malaria when *P. falciparum* malaria should be non-detected. The second row considers the presence (1) of *P. falciparum* malaria. Of the 1729 cases, 1615 were correctly classified as presence (1) of *P. falciparum* malaria, while the remaining (114) were wrongly classified as absence of *P. falciparum* malaria when *P. falciparum* malaria should be detected (Table 6).

Prediction

|  | Absence (0) | Presence (1) | Row total |
| --- | --- | --- | --- |
| Calculated Absence (0) | 198 | 387 | 585 |
| Presence (1) | 114 | 1615 | 1729 |
| Colum total | 312 | 2002 | 2314 |

Table S5: Confusion matrix for the CART model for *P. falciparum* malaria presence in Africa.

For the GLM model, the confusion matrix shows that of the 585 cases, 149 were correctly classified as absence of *P. falciparum* malaria, while the remaining (436) were wrongly classified as presence of *P. falciparum* malaria when *P. falciparum* malaria should be non-detected. For the presence (1) of *P. falciparum* malaria, of the 1729 cases, 1637 were correctly classified as presence of *P. falciparum* malaria, while the remaining (92) were wrongly classified as absence of *P. falciparum* malaria when *P. falciparum* malaria should be detected (Table 7).

Prediction

|  | Absence (0) | Presence (1) | Row total |
| --- | --- | --- | --- |
| Calculated Absence (0) | 149 | 436 | 585 |
| Presence (1) | 92 | 1637 | 1729 |
| Colum total | 241 | 2073 | 2314 |

Table S6: Confusion matrix for the GLM model for *P. falciparum* malaria presence in Africa.

|  | Model | |
| --- | --- | --- |
| Metric | CART | GLM |
| Accuracy | 78.4 | 77 |
| Precision | 81 | 79 |
| Recall | 93 | 95 |
| F1-score | 87 | 86 |

Table S7: Accuracy, precision, recall, and F1-score results from the performance assessment to the CART and GLM models for *P. falciparum* presence in Africa. Values are expressed in percentages.

Even though our models show high accuracy, both of them overestimated the number of false negatives, especially the GLM model. This is very common when there is a dominant class which in this case is the true negatives (absence) which in our data set represent the high number of zeros. In this situation, it is recommended to perform further tests such as the calculation of the model precision, the model recall, and the F1-score.

**Model performance assessment for** *Plasmodium vivax* **malaria presence in Asia.**

We built confusion matrices for our CART model (Table S8) and for our GLM model (Table S9). The first row of the CART confusion matrix shows the absence (0) of *P. vivax* malaria. Of the 489 cases, 366 were correctly classified as absence of *P. vivax* malaria, while the remaining (123) were wrongly classified as presence of *P. vivax* malaria. The second row considers the presence (1) of *P. vivax* malaria. Of the 375 cases, 287 were correctly classified as presence of *P. vivax* malaria, while the remaining (88) were wrongly classified as absence of *P. vivax* malaria (Table S8).

Prediction

|  | Absence (0) | Presence (1) | Row total |
| --- | --- | --- | --- |
| Calculated Absence (0) | 366 | 123 | 489 |
| Presence (1) | 88 | 287 | 375 |
| Colum total | 454 | 410 | 864 |

Table S8: Confusion matrix for the CART model for the prediction of *P. vivax* presence in Asia. The confusion matrix is based on 864 observations.

For the GLM model, the confusion matrix shows that of the 489 cases, 397 were correctly classified as absence of *P. vivax* malaria, while the remaining (92) were wrongly classified as presence of *P. vivax* malaria when *P. vivax* malaria should be non-detected. For the presence (1) of *P. vivax* malaria, of the 375 cases, 236 were correctly classified as presence of *P. vivax* malaria, while the remaining (139) were wrongly classified as absence of *P. vivax* malaria when *P. vivax* malaria should be detected (Table S9).

Prediction

|  | Absence (0) | Presence (1) | Row total |
| --- | --- | --- | --- |
| Calculated Absence (0) | 397 | 92 | 489 |
| Presence (1) | 139 | 236 | 375 |
| Colum total | 536 | 328 | 864 |

Table S9: Confusion matrix for the GLM model for the prediction of *P. vivax* malaria presence in Asia. The confusion matrix is based on 864 observations.

|  | Model | |
| --- | --- | --- |
| Metric | CART | GLM |
| Accuracy | 76 | 73 |
| Precision | 70 | 72 |
| Recall | 76.5 | 63 |
| F1-score | 73 | 67 |

Table S10: Accuracy, precision, recall, and F1-score results from the performance assessment for *P. vivax* presence in Asia. Values are expressed in percentages.

# References

1. Goutte, C. & Gaussier, E. A probabilistic interpretation of precision, recall and f-score, with implication for evaluation (ed. Losada, D.E. & Fernandez-Luna, J.M.) 345–359 (Springer, 2015)
2. Irmanita, R., Prasetiyowati, S. S., Sibaroni, Y., et al. Classification of Malaria Complication Using CART (Classification and Regression Tree) and Naïve Bayes. *RESTI Journal*. **5**, 10–16. <https://doi.org/10.29207/resti.v5i1.2770> (2021).
